# Epigallocatechin-3-Gallate Alleviates Manganese-Induced Cardiac Injury in Common Carp via Suppression of the Integrated Stress Response–Inflammation Axis

**DOI:** 10.3390/biology15201866

**Published:** 2026-10-16

**Authors:** Yan Wang, Hao Wang, Shi Li, Yuejia Han, Cao Yu, Yuhao Liu

**Affiliations:** 1 School of Public Health, Beihua University, Jilin 132013, China; 2 College of Animal Science and Technology, Northeast Agricultural University, Harbin 150030, China; 3 State Key Laboratory for Diagnosis and Treatment of Severe Zoonotic Infectious Diseases, Institute of Zoonosis, College of Veterinary Medicine, Jilin University, Changchun 130062, China; 4 Key Laboratory for Zoonosis Research of the Ministry of Education, Institute of Zoonosis, College of Veterinary Medicine, Jilin University, Changchun 130062, China

**Keywords:** excess manganese, epigallocatechin-3-gallate, common carp heart, integrated stress response, inflammation

## Abstract

Manganese (Mn) pollution in aquatic environments poses threats to fish health, yet the molecular mechanisms of Mn-induced cardiac injury remain poorly understood. Epigallocatechin-3-gallate (EGCG), the major bioactive catechin in green tea, exhibits potent antioxidant and anti-inflammatory properties. Bioinformatics revealed overlap between Mn toxicity targets and EGCG-modulated genes, enriched in oxidative stress, ER stress and inflammation-related mechanisms. Therefore, this study aimed to investigate the molecular mechanisms by which Mn exposure induces cardiotoxicity in common carp and to evaluate whether dietary EGCG supplementation confers protection against cardiac injury, with particular focus on the roles of integrated stress response (ISR) and inflammation. Carp were exposed to Mn^2+^ with or without dietary EGCG. We found that Mn exposure resulted in myocardial histological structural damage, elevated serum cardiac injury markers, and reduced myocardial ATPase activities, indicating cardiac injury at the structural and biochemical levels. Mechanistically, Mn-induced oxidative stress (decreased *SOD1*, *CAT*, *NRF2*; increased MDA) was associated with ER stress and ISR-related transcript changes (upregulated *HSPA5*, *DDIT3*, *eiF2AK4*, *atf4b*) and triggered inflammatory responses (increased IL-1β, IL-6, IFN-γ, iNOS/NO), alongside upregulated inflammatory signaling nodes (NF-κB, STAT3, JUN, MAPK1, MAPK14b). EGCG supplementation significantly reversed these pathological changes. Importantly, ISRIB treatment in EPC epithelial cells and H9c2 rat cardiac-derived myoblasts attenuated Mn-induced inflammatory gene expression, providing supporting mechanistic evidence that ISR-associated signaling may contribute to inflammation, although these in vitro findings do not directly confirm the proposed mechanism in carp cardiomyocytes. Taken together, these findings support a proposed mechanistic framework in which waterborne Mn exposure is associated with oxidative stress, ER stress, and ISR-related signaling, together with inflammatory responses and cardiac injury in common carp, rather than a conclusively demonstrated causal pathway. Dietary EGCG supplementation attenuated these Mn-associated responses and was associated with reduced cardiac injury. Our work provide a new mechanistic way of thinking for understanding heavy metal-caused cardiac injury in fish and improving the current application potential of EGCG as an experimental dietary intervention for aquatic pollution stress management.

## Simple Summary

Manganese (Mn) pollution in aquatic environments can harm fish, but how it induces cardiac injury remains unclear. We exposed common carp to manganese with or without dietary epigallocatechin-3-gallate (EGCG), a major green tea compound. Mn caused cardiac injury, oxidative stress, endoplasmic reticulum stress, ISR-associated changes, and inflammation. EGCG supplementation largely reversed these changes. Cell experiments using ISRIB further supported a link between ISR-associated signaling and inflammation. These findings suggest that EGCG may protect fish hearts against Mn toxicity by suppressing the ISR–inflammation axis, providing preliminary experimental evidence for the potential protective effects of dietary EGCG in fish.

## 1. Introduction

With the acceleration of global industrialization and urbanization, large amounts of heavy metal pollutants are discharged into aquatic environments, posing a serious threat to the health of aquatic ecosystems. Although manganese (Mn) is an essential trace element for organisms, excessive Mn discharge causes water pollution. Notably, in aquaculture water bodies, manganese and zinc are the most abundant heavy metals in sediments, accounting for 68–78% of the total heavy metal content [1]. Continuous inputs from industrial wastewater discharge, mining activities, and aquaculture feed lead to rising Mn concentrations in water. Fish, as key components of aquatic ecosystems, are highly sensitive to changes in the water environment. Studies confirm that Mn significantly bioaccumulates in fish tissues such as gills, liver, and kidneys. However, the toxic mechanisms of Mn on fish hearts remain poorly understood. The common carp (Cyprinus carpio), a globally important economic aquaculture species and an indicator organism for environmental pollution monitoring, has cardiac tissue as a target organ for heavy metal attack [2,3,4]. Heart health is crucial for the survival rate, growth rate, welfare, and flesh quality of economic fish. Therefore, in-depth investigation into the molecular mechanisms of cardiac injury induced by Mn exposure in carp holds significant scientific importance for assessing the aquatic ecological risks of waterborne Mn pollution and ensuring the healthy development of the aquaculture industry.

Mn exposure can induce tissue damage through multiple pathways, including oxidative stress and endoplasmic reticulum stress. Oxidative stress is a pathological state characterized by an imbalance between the production of reactive oxygen species (ROS) and the antioxidant defense system, leading to lipid peroxidation and protein and DNA damage. Studies have shown that Mn exposure induces oxidative stress responses in fish, manifested as altered antioxidant enzyme activities and elevated ROS levels. Besides oxidative stress, endoplasmic reticulum (ER) stress is another important pathway for heavy metal-induced cell damage. ER stress refers to the abnormal accumulation of unfolded or misfolded proteins within the ER lumen, triggering the unfolded protein response (UPR) to restore protein homeostasis. Notably, oxidative stress and ER stress are not isolated events; rather, they can converge on a highly conserved intracellular signaling pathway: the integrated stress response (ISR). The ISR is an evolutionarily conserved cellular homeostatic mechanism activated by various internal and external stressors [5,6]. The core mechanism of the ISR involves the global suppression of protein synthesis and the initiation of downstream biological signals when cells perceive adverse signals such as amino acid deprivation, ER stress, and oxidative stress. The ISR serves as a signaling hub for oxidative stress and ER stress, integrating diverse cellular damage signals into a unified transcriptional–translational regulatory response. However, whether Mn can induce the ISR in fish has not yet been reported.

Recent studies have revealed a close regulatory link between the ISR and inflammatory responses. In tissue-resident memory CD4^+^ T cells, the ISR pathway maintains immune homeostasis by inhibiting the translation of pro-inflammatory cytokine mRNAs. In aquatic animals, excessive Mn treatment reduces growth performance and induces oxidative stress and inflammatory tissue damage in the liver of largemouth bass (Micropterus salmoides) [7]. Therefore, inflammation may be a significant mechanism in the toxic effects of Mn on carp. The activation of inflammatory signaling pathways is regulated by multiple core transcription factors and kinases, such as NF-κB, STAT3, JUN, and members of the MAPK family. Notably, while the ISR suppresses global protein translation, it selectively upregulates the expression of transcription factors like ATF4. Whether this upregulation can promote the expression of the aforementioned core node genes in inflammatory signaling upon Mn exposure remains to be elucidated. Clarifying the regulatory relationship between the ISR and these core nodes of inflammatory signaling is of great significance for a deeper understanding of the initiation and maintenance mechanisms of inflammatory responses under stress conditions.

Epigallocatechin-3-gallate (EGCG) is the most abundant and biologically active catechin in green tea. EGCG possesses significant pharmacological activities, including antioxidant, anti-inflammatory, and anti-ER stress properties. In terms of cardioprotection, EGCG can alleviate myocardial fibrosis and improve cardiac function after myocardial infarction by inhibiting TGF-β2- and IL-1β-induced endothelial-to-mesenchymal transition [8]; it also reduces cardiac damage and improves myocardial function in mice with myocardial infarction by inhibiting oxidative stress and inflammation-related factors [9]. Furthermore, EGCG has been shown to effectively block the vicious cycle between ROS and inflammation during myocardial ischemia–reperfusion injury by scavenging ROS and inhibiting the TLR4 signaling pathway, thereby improving cardiac function and reducing scar formation [10]. In terms of inhibiting ER stress, EGCG can alleviate ER stress via the PERK-eIF2α-ATF4 pathway, mitigating cadmium-induced developmental toxicity in Caenorhabditis elegans [11]. Therefore, we attempted to supplement feed with EGCG to explore whether it could alleviate Mn-induced cardiac injury in carp and to elucidate its molecular mechanisms.

Despite evidence linking Mn exposure to oxidative stress and inflammation in fish, it remains unresolved whether ISR-associated signaling contributes to Mn-induced cardiac inflammatory responses and whether EGCG-associated protection is accompanied by attenuation of these responses. In this study, we utilized the Comparative Toxicogenomics Database (CTD) to retrieve Mn toxicity-related targets and the BATMAN-TCM 2.0 database to predict EGCG targets. Functional enrichment analysis of the overlapping targets revealed that both Mn toxicity targets and EGCG-modulated genes were significantly enriched in pathways related to oxidative stress, inflammatory response, and ER stress, with numerous intersecting targets. Based on these bioinformatics findings, we established a carp model with EGCG supplementation and/or Mn exposure to investigate the molecular mechanisms by which EGCG alleviates Mn-caused cardiac injury, focusing on the ISR mediated by oxidative stress and ER stress, as well as inflammatory responses. The results of this study aim to elucidate the novel mechanisms and core pathways of Mn-induced cardiac injury in carp, providing a crucial theoretical basis and experimental support for the development of EGCG for the health protection of fish in heavy metal-polluted waters and as an experimental anti-stress dietary intervention in aquaculture.

In summary, we hypothesized that ISR-associated signaling may contribute to Mn exposure-induced common carp cardiac injury, and that dietary EGCG alleviates this damage by inhibiting these pathways. The ISR was specifically chosen because it serves as a shared downstream signaling hub integrating oxidative stress and ER stress, is evolutionarily conserved but unexplored in Mn-exposed fish, is mechanistically linked to inflammation via ATF4, and can be pharmacologically targeted by ISRIB (an ISR inhibitor), allowing direct causal testing. This hypothesis was tested using a 60-day Mn exposure model with EGCG supplementation and ISRIB-based cell experiments.

## 2. Materials and Methods

### 2.1. Animal Model

All animal experimental procedures complied with the regulations of the Animal Ethics Committee of Northeast Agricultural University (Approval No.: NEAUEC20240229). Healthy common carp (*Cyprinus carpio*) (initial body weight 76.88 ± 4.10 g) used in this experiment were provided by the Heilongjiang River Fisheries Research Institute, Chinese Academy of Fishery Sciences (Harbin, China). The fish were acclimatized in a laboratory recirculating water culture system for 2 weeks under the following conditions: water temperature 24 ± 2 °C, pH 7.2 ± 0.3, dissolved oxygen ≥ 6.0 mg/L, and a 12 h light/12 h dark photoperiod. During the experiment, fish were fed twice daily with commercial carp compound feed at 3% of body weight, and one-third of the total water volume was replaced daily. After acclimatization, a total of 108 carp were randomly divided into 4 groups (3 replicates per group, 9 fish per replicate). The formal experiment lasted for 60 consecutive days, and the Mn exposure treatment was maintained throughout the entire experimental period. The grouping settings were as follows: Control group (Con) reared in normal aerated tap water; Mn treatment group (Mn) continuously exposed to 3.5 mg/L (based on Wang et al. (2023) and the measured environmental concentration in Islam (2023) [11,12]); and EGCG supplementation group (EG) receiving a basal diet supplemented with 75 mg/kg EGCG (Epigallocatechin-3-gallate, purity > 98%, Macklin Biochemical Technology Co., Ltd., Shanghai, China; these are theoretical offered amounts, not measurements of consumption, absorbed dose or bioavailability). The EGCG dose was consistent with our previous study [7]. The EGCG-supplemented diet was prepared as follows: The basal diet consisted of commercial carp compound feed. EGCG was added by sequential dilution to reach a target concentration of 75 mg/kg feed, with repeated and thorough mixing throughout the preparation process to ensure homogeneity. After preparation, all feeds were sealed, protected from light, and stored at 4 °C and dry conditions to prevent oxidative degradation of EGCG. The storage duration shall not exceed 3 months. The actual EGCG content in the feed was verified by HPLC to be consistent with the intended dose of 75 mg/kg, and the mixing homogeneity was satisfactory, ensuring stable intervention doses and reliable experimental results. At the initial mean body weight of 76.88 g, this corresponds to approximately 5.766 mg EGCG per fish/day. The combined group (Mn+EG) was exposed to 3.5 mg/L Mn^2+^ in water, with the diet containing 75 mg/kg EGCG, twice daily at 08:00 and 18:00. The Mn and Mn+EG groups had one-third of the water replaced daily with fresh water supplemented with MnCl_2_·4H_2_O to maintain the Mn^2+^ concentration [13]. To ensure stable Mn exposure concentration throughout the experiment, the water Mn^2+^ concentration was monitored daily (total 60 days) using atomic absorption spectrophotometry, and the detection result was 3.51 ± 0.09 mg/L. This concentration was confirmed to be sublethal, as no mortality occurred in any group during the 60-day exposure. Throughout the 60-day exposure, water temperature, pH and dissolved oxygen were maintained at 24 ± 2 °C, 7.2 ± 0.3 and ≥6.0 mg/L, respectively, by the recirculating culture system. The total hardness of the water in this system was 241 mg/L as CaCO_3_, consistent with our previous study using the same recirculating water system [14]. The Con and EG groups received equal volumes of aerated tap water daily. The experiment lasted for 60 days. Feeding, swimming status, and behavioral changes in fish in each group were observed and recorded daily throughout the 60-day experimental period. During the 60-day experimental period, feeding and growth data were recorded for each group, and feed intake (FI), weight gain rate (WGR), survival rate (SR), and the feed conversion ratio (FCR) were calculated.

### 2.2. Sample Collection

After the experiment, all carp were fasted for 12 h and anesthetized with 100 mg/L MS-222 (Sigma-Aldrich, St. Louis, MO, USA), and blood was collected from the caudal vein along the midline of the caudal peduncle. Blood samples were allowed to clot at 4 °C for 2 h and then centrifuged at 4000 rpm for 5 min to separate serum, which was stored at −80 °C for later use. After blood collection, heart tissues were rapidly dissected. Part of the tissue was fixed in 4% paraformaldehyde for histopathological observation, and another part was rapidly frozen in liquid nitrogen and stored at −80 °C for subsequent molecular biology analyses. For all serum biochemical, tissue biochemical, histopathological, and molecular analyses, two fish were randomly sampled from each replicate tank, yielding six fish per group (*n* = 6). Each individual fish was treated as one biological replicate, and no samples were pooled.

### 2.3. Serum Biochemical Index Detection

Blood samples were allowed to stand for 30 min and centrifuged at 4000 r/min for 15 min to obtain the supernatant. The obtained serum was stored in a refrigerator and sent to the Third Affiliated Hospital of Heilongjiang University of Chinese Medicine (Harbin, China) for the detection of aspartate aminotransferase (AST), lactate dehydrogenase (LDH), and α-hydroxybutyrate dehydrogenase (α-HBDH) using a biochemical analyzer (TBA-2000FR, Toshiba, Tokyo, Japan).

### 2.4. Cardiac Antioxidant Index Detection

All kits were purchased from Nanjing Jiancheng Bioengineering Institute (Nanjing, China) for measuring the following indicators, namely serum creatine kinase (CK, kit no. A032-1), four ATPases (Na^+^/K^+^-ATPase, Mg^2+^-ATPase, Ca^2+^-ATPase, and Ca^2+^/Mg^2+^-ATPase, kit no. A016-2), two antioxidant enzymes (CAT, kit no. A007-1; SOD, kit no. A001-3), and NO (kit no. A012-1), following the kit instructions. A microplate reader (Molecular Devices Co. Ltd., Shanghai, China) was used to detect the absorbance for all the above indicators.

### 2.5. Cardiac Ultrastructure Examination

Heart tissues fixed in 2.5% glutaraldehyde phosphate buffer were first thoroughly rinsed with the same buffer to remove residual fixative, then post-fixed in 1% osmium tetroxide to enhance membrane contrast. Subsequently, tissue blocks were dehydrated through a graded ethanol series (30% to 100%), gradually transferred into propylene oxide, and infiltrated with epoxy resin embedding medium. Polymerization was completed by gradual heating from 37 °C to 60 °C. The polymerized blocks were sectioned into ultrathin sections of approximately 70 nm thickness using an ultramicrotome. Sections were picked up onto copper grids and sequentially double-stained with uranyl acetate and lead citrate to enhance electron scattering. Finally, the grids were placed in a transmission electron microscope (GEM-1200ES, JEOL Ltd., Tokyo, Japan) sample chamber and observed at an accelerating voltage of 80 kV. Representative areas were selected for focused imaging, and high-resolution ultrastructural images were captured with a camera for subsequent analysis.

### 2.6. H&E Staining and Cardiac Pathological Scoring

Carp heart tissue blocks fixed in 4% paraformaldehyde solution were dehydrated, embedded in paraffin, and cut into 5–6 μm thick sections. Sections were then deparaffinized with ethanol and xylene, stained with hematoxylin and eosin (Servicebio Co. Ltd., Wuhan, China), and sealed with neutral gum. Stained slides were scanned using a digital slide scanning system (Shandong Zhiying Medical Technology Co., Ltd., Jinan, China), and the microstructure of the tissue was observed using ZYF Viewer software (version 1.0.0.0, Shandong Zhiying Medical Technology Co., Ltd., Jinan, China). Histopathological evaluation was performed on three sections per group. For each section, ten randomly selected microscopic fields were examined by an investigator blinded to the group allocation, and representative images were selected for presentation. Cardiac pathological changes were evaluated semi-quantitatively in a blinded manner based on the following parameters: (1) cardiomyocyte degeneration (vacuolization, loss of cross-striations, fragmentation); (2) cardiomyocyte necrosis; (3) inflammatory cell infiltration; (4) myocardial fibrosis; (5) myocardial disarray; (6) vascular congestion/hemorrhage; (7) interstitial edema; and (8) myocardial hypertrophy. Each parameter was graded on a scale of 0 to 3 (0 = normal/absent; 1 = mild; 2 = moderate; 3 = severe), and the total score was calculated as the sum of all parameters.

### 2.7. Acquisition of Potential Target Genes for EGCG and Mn

Potential EGCG-associated candidate targets were retrieved from the BATMAN-TCM 2.0 database using “epigallocatechin-3-gallate” as the query, whereas Mn-associated candidate targets were retrieved from the Comparative Toxicogenomics Database (CTD) using “manganese” as the query. The databases were accessed on 2026.8.26. After the preprocessing criteria, the overlap between the EGCG-associated and Mn-associated gene sets was defined as the set of overlapping candidate targets for subsequent bioinformatics analyses.

### 2.8. Enrichment Analysis

The String database (https://string-db.org) and the DAVID database (https://davidbioinformatics.nih.gov/) were utilized for GO (BP, CC, MF) and KEGG pathway enrichment analysis of the obtained EGCG potential target genes and the intersecting genes of EGCG and Mn, with this undertaken to investigate potential pathways affected by the core targets. In the String database, all gene names were entered into the search box, Danio rerio was selected as the species, and KEGG and GO enrichment results were obtained. On the DAVID platform, official gene symbols were used as input IDs, common carp (or Danio rerio for broader annotation) was selected as the species, and GOTERM_BP_DIRECT, GOTERM_CC_DIRECT, GOTERM_MF_DIRECT, and KEGG_PATHWAY were selected for enrichment analysis. The results were then visualized. It should be noted that common carp gene symbols were used as conserved cross-species identifiers for STRING and DAVID analyses, with Danio rerio selected as the reference species because of its well-annotated genome and close phylogenetic relationship with common carp. This approach infers conserved pathway-level associations rather than establishing one-to-one ortholog relationships. Given the fourth-round whole-genome duplication in common carp, duplicated gene pairs were not systematically resolved, and the results should be interpreted as pathway-level predictions that require experimental validation in carp.

Functional enrichment analyses were performed using STRING (v 12.0/2026.08.26) and DAVID (v 2026_1/2026.08.26). GO enrichment, including biological process (BP), cellular component (CC), and molecular function (MF), and KEGG pathway enrichment analyses were conducted for the EGCG-associated candidate genes and the overlapping candidate genes. In STRING, gene symbols were used as input and Danio rerio was selected as the reference species because common carp was not available as a species option in the analysis used in this study. In DAVID, official gene symbols were used as identifiers, with common carp selected where supported and Danio rerio used as a reference species when broader functional annotation was required. GOTERM_BP_DIRECT, GOTERM_CC_DIRECT, GOTERM_MF_DIRECT, and KEGG_PATHWAY were included in the DAVID enrichment analysis.

The default species-specific background provided by STRING and DAVID was used for the enrichment analyses, and no customized background gene set was specified. Enrichment significance was evaluated using multiple-testing-adjusted statistics provided by the respective databases, with an FDR < 0.05 considered statistically significant. For DAVID analyses, Benjamini-adjusted *p* values were used to account for multiple comparisons. Overlapping genes between the EGCG-associated and Mn-associated candidate gene sets were retained as candidate targets for subsequent enrichment analyses.

No systematic one-to-one orthology mapping between common carp and zebrafish was performed. Instead, common carp gene symbols were queried against the reference annotations based on conserved gene identifiers. Therefore, analyses based on the zebrafish reference should be interpreted as cross-species, pathway-level predictions rather than direct evidence of one-to-one orthologous relationships or experimentally validated molecular functions in common carp. Given the whole-genome duplication history of common carp and the presence of duplicated gene pairs, potential paralog-specific differences were not systematically resolved in the present analysis. Accordingly, all database-derived targets and enrichment results were considered hypothesis-generating and require experimental validation in common carp.

### 2.9. Cell Culture and Treatment

Epithelioma papulosum cyprini (EPC) cells serves as a model for investigating common carp physiology. We thawed previously liquid nitroge-preserved EPC cells and H9c2 cells and cultured. Briefly, once revived, the EPC cells were seeded into T-25 flasks, with each flask containing 4 mL of culture medium (89% M199 (Biosharp, BL309A, Hefei, China), 10% fetal bovine serum (Biological Industries, Beit Haemek, Israel), and 1% penicillin–streptomycin mixture (Servicebio, Wuhan, China)). The flasks were then incubated in a humidified chamber set at 27 °C with 5% CO_2_. H9c2 cells were cultured in H9c2(2-1) cell-specific medium (Procell Life Science Co., Ltd., Wuhan, China) at 37 °C in a humidified atmosphere of 5% CO_2_. Following 4 passages, the cells were divided into 6 groups, including the Control group, Mn group, EGCG group, Mn+EGCG group, ISRIB group, and Mn+ISRIB group. The Mn, Mn+EGCG, and Mn+ISRIB groups were treated with 150 µM Mn for EPC cells [7] and 200 µM Mn for H9c2 cells. The EGCG and Mn+EGCG groups were treated with 250 µM EGCG for EPC cells [7] and 20 µM EGCG for H9c2 cells [15]. The ISRIB and Mn+ISRIB groups were treated with 200 nM ISRIB [16]. After a 24 h treatment, cells were used for qRT-PCR detection.

### 2.10. Quantitative Real-Time PCR

Gene sequences were obtained from GenBank. β-actin served as the reference gene. Primer information for our experiment is shown in Tables S1 and S2. Primers were synthesized by Sangon Bioengineering Co., Ltd. (Shanghai, China). Primer amplification efficiencies were evaluated using standard curves generated from serial dilutions of cDNA and were within the acceptable range of 90–110%. The synthesized primers were briefly centrifuged, and then DNase/RNase-free water (SolarBio, Beijing, China) was added to the EP tubes containing the primers according to the instructions to prepare working solutions. The working solutions were mixed and stored in a −20 °C freezer for later use.

Ground tissue samples or cell samples were mixed with 1 mL of Trizol (Takara, Kusatsu, Shiga, Japan), and the tubes were placed in a low-temperature grinder (Hangzhou Suizhen Biotechnology Co., Ltd., Hangzhou, China) pre-cooled to 4 °C for sample grinding. After sequentially adding chloroform, isopropanol, and 75% DEPC ethanol to the tubes, total RNA was extracted from each heart sample. The concentration of the obtained total RNA was measured using a micro-spectrophotometer (Hangzhou Aosheng Instruments Co., Ltd., Hangzhou, China). The OD 260/280 ratio (greater than 1.9 and less than 2.1) met the total RNA purity requirements. Reverse transcription for mRNA was performed in a 20 μL system according to the instructions of the FastKing cDNA First Strand Synthesis Kit (kit no. KR180123, Tiangen Bio., Beijing, China). The LightCycler^®^480 Real-Time PCR System (Roche, Basel, Switzerland) was used.

Each biological sample was analyzed in three technical replicates. No-template controls (NTCs), in which nuclease-free water was substituted for the cDNA template, and no-reverse-transcription (no-RT) controls were included to monitor potential reagent contamination and genomic DNA contamination, respectively. No specific amplification was detected in these controls. Following amplification, melting-curve analysis was performed to verify amplification specificity, and only reactions showing a single specific melting peak were included in the analysis. Reactions exhibiting abnormal amplification curves, nonspecific melting peaks, or substantial inconsistency among technical replicates were excluded from subsequent analysis.

The stability of β-actin under the experimental conditions was assessed by comparing its raw Ct values among the control, Mn, EGCG, and Mn + EGCG groups, with no significant differences observed among treatments (*p* > 0.05). Relative mRNA expression levels were normalized to β-actin and calculated using the 2^−ΔΔCt^ method.

### 2.11. ELISA Kit Assay

The protein levels of p-eIF2α, eIF2α, ATF4, CHOP, PERK, GCN2, NF-κB, STAT3, MAPK1, MAPK14B, and JUN were measured using ELISA kits of rat (purchased from Lupu Technology Co., Ltd., Shanghai, China). All experimental procedures were performed strictly according to the instructions provided by the kit manufacturer. Briefly, cells were washed with PBS and lysed in ice-cold lysis buffer supplemented with protease and phosphatase inhibitors. Lysates were centrifuged at 12,000× *g* for 15 min at 4 °C, and the supernatants were collected. Total protein concentration was determined by the BCA assay. Equal amounts of protein samples and standards were added to the pre-coated ELISA plates and incubated as specified. After washing, detection antibody and HRP-conjugated reagent were added sequentially, followed by TMB substrate for color development. The reaction was stopped with stop solution, and absorbance was measured at 450 nm. Protein concentrations were calculated from the standard curve and normalized to total protein content. The ratio of p-eIF2α/eIF2α was also calculated. These protein-level measurements were performed by ELISA in H9c2 cells, not in carp heart tissue.

### 2.12. Multiple Comparison Correction

Multiple-testing corrections were applied at three distinct levels, each restricted to its own scope. First, for comparisons among the four groups for each individual endpoint, one-way ANOVA, followed by Tukey’s post-hoc test, was used to control the family-wise error rate within that endpoint. Second, for the GO and KEGG enrichment analyses, multiple-testing-adjusted statistics provided by the STRING and DAVID databases were used, with an adjusted FDR < 0.05 considered statistically significant; for DAVID analyses, Benjamini-adjusted *p* values were used. These database-derived corrections were applied only within the enrichment analyses and were not applied to the experimental endpoints. Third, no formal correction was applied across the different experimental endpoints, and no single procedure controlled all multiple testing in this study; this is acknowledged as a limitation in Section 4.4.

### 2.13. Statistical Analysis

All statistical analyses were performed using IBM SPSS Statistics 23 (IBM Corp., Armonk, NY, USA). For in vivo data, the tank was the experimental unit (*n* = 3 tanks per group), and individual fish (*n* = 6 per group) were treated as subsamples. For in vitro data, *n* = 3 independent cell cultures. The sample size was determined according to the routine practice of our research team and previously published studies with similar experimental designs (e.g., [7,13,17]), which indicated that three replicate tanks per group with six sampled fish per group provide sufficient replication to detect biologically meaningful differences in the primary endpoints. Normality was assessed using the Shapiro–Wilk test and homogeneity of variance using Levene’s test. Differences among groups were analyzed by one-way ANOVA followed by Tukey’s multiple-comparison post-hoc test, which controls the family-wise error rate within each endpoint. No outliers were excluded from the analysis. Different lowercase letters indicate significant differences between groups (*p* < 0.05). Data are expressed as mean ± SD and were plotted using GraphPad Prism 8.0 (San Diego, CA, USA).

## 3. Results

### 3.1. EGCG Alleviated Mn-Induced Cardiac Injury in Carp

**Figure 1:**
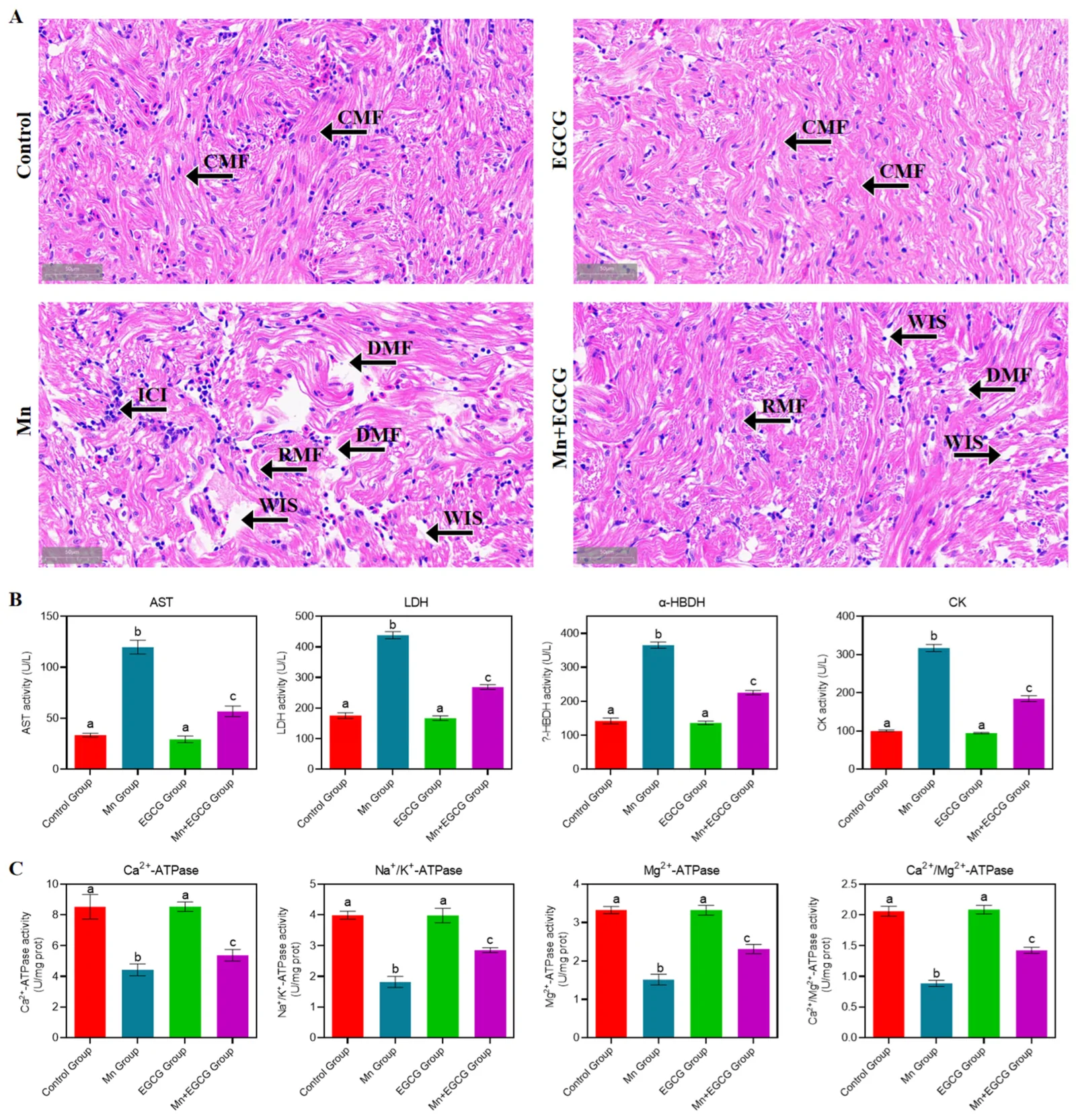
EGCG alleviated Mn-induced cardiac damage in carp. (**A**) Representative H&E staining images of carp heart tissues showing myocardial fiber morphology (Scale bar = 50 μm). MF: myocardial fibers; RMF: disorganized myocardial fibers; DMF: broken myocardial fibers; WIS: widened intermyocardial spaces; ICI: inflammatory cell infiltration. (**B**) Serum levels of cardiac injury markers AST, LDH, CK, and α-HBDH in different groups. (**C**) Activities of Na^+^/K^+^-ATPase, Ca^2+^-ATPase, Mg^2+^-ATPase, and Ca^2+^/Mg^2+^-ATPase in carp heart tissues. For each group, 2 fish were randomly selected from each of the 3 replicate tanks (6 fish per group). Data are expressed as mean ± SD (*n* = 3 tanks per group). Different lowercase letters indicate significant differences between groups (*p* < 0.05).

For growth performance (Table 1), we found that the WG and FCR of the Control, Mn, and Mn+EGCG groups showed no significant differences, indicating that low-concentration Mn did not markedly affect the growth of common carp. The EGCG group had higher WG than the other three groups, suggesting that EGCG can effectively improve the growth performance of common carp, but this effect was lost upon Mn exposure. We then investigated whether excessive Mn could damage the carp heart and whether dietary EGCG supplementation could provide relief, using cardiac morphology and cardiac injury-related markers. H&E staining results are shown in Figure 1A. The control and EGCG groups showed regularly arranged, dense, and structurally intact myocardial fibers (MFs). The Mn group exhibited tissue damage, including disorganized myocardial fibers (RMFs), broken myocardial fibers (DMFs), widened intermyocardial spaces (WISs), and inflammatory cell infiltration (ICI). The Mn+EGCG group showed RMF, DMF, and WIS with less severity than the Mn group. We further performed cardiac pathological scoring and found that the pathological score of the Mn+EGCG group was significantly lower than that of the Mn group (Figure S1). This confirmed at the morphological level that EGCG supplementation effectively alleviated Mn-induced myocardial structural damage. During the 60-day exposure, no mortality was observed in any group. Compared with the Con group, fish in the Mn group exhibited reduced swimming activity and feeding motivation, whereas these behavioral changes were partially alleviated in the Mn+EG group, whose activity and feeding levels were intermediate between those of the Con and Mn groups.

AST, LDH, CK, and α-HBDH are markers of myocardial injury. Detection of these four serum indicators in carp revealed that Mn exposure elevated AST, LDH, CK, and α-HBDH, while EGCG supplementation reversed these changes, further confirming that EGCG supplementation effectively alleviated Mn-induced myocardial injury in carp (Figure 1B).

Na^+^/K^+^-ATPase, Ca^2+^-ATPase, Mg^2+^-ATPase, and Ca^2+^/Mg^2+^-ATPase are key enzymes on cardiomyocyte membranes that maintain ion homeostasis and energy metabolism. Changes in their activities are biochemical indicators of myocardial stress and cellular injury, although they do not directly reflect cardiac performance. These results provide further biochemical evidence that EGCG alleviates Mn-induced myocardial injury. Specifically, as shown in Figure 1C, compared with the control and EGCG groups, the activities of Na^+^/K^+^-ATPase, Ca^2+^-ATPase, Mg^2+^-ATPase, and Ca^2+^/Mg^2+^-ATPase in the heart tissues of the Mn-treated group were decreased. Compared with the Mn group, the activities of these four ATPases in the Mn+EGCG combined treatment group were increased.

### 3.2. Integrated Stress Response and Inflammation-Related Genes Are Potential Regulatory Targets of EGCG

**Figure 2:**
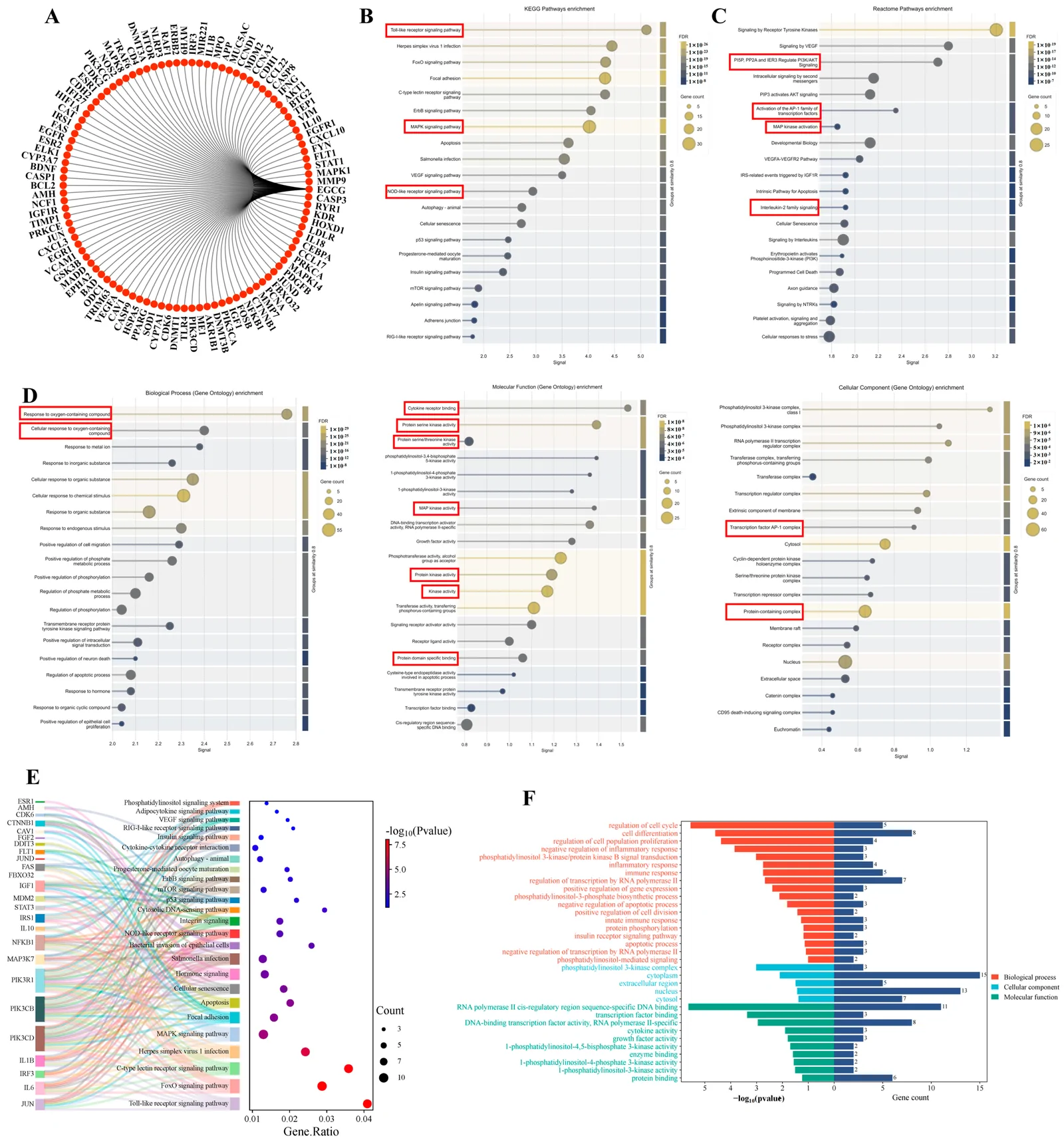
Integrated stress response and inflammation-related genes are potential regulatory targets of EGCG. (**A**) Venn diagram showing the 124 potential targets of EGCG predicted by the BATMAN-TCM 2.0 database. (**B**) KEGG pathway enrichment analysis of EGCG targets using the String database. (**C**) Reactome pathway enrichment analysis of EGCG targets. (**D**) Gene Ontology (GO) enrichment analysis of EGCG targets (BP: Biological Process; CC: Cellular Component; MF: Molecular Function) using the String database. (**E**) KEGG pathway enrichment analysis of EGCG targets using the DAVID database. (**F**) GO enrichment analysis of EGCG targets using the DAVID database. The red boxes highlights KEGG pathways or GO terms related to oxidative stress, endoplasmic reticulum stress, and inflammation.

To explore the potential mechanisms by which EGCG alleviates Mn toxicity, we analyzed the potential targets of EGCG. We obtained 124 targets, including inflammation-related genes (*IL18*, *CCL17*, *TLR4*, *IL1B*, *TNF*, *IL6*, etc.), oxidative stress-related genes (*SOD1*, *CAT*, *NCF1*, *MPO*, *NFE2L2*, etc.), and ER stress-related genes (*HSPA5*, *DDIT3*, *MAPK8*, *MAPK14*, etc.) (Figure 2A and Table S3). KEGG pathway enrichment analysis using the String database showed that the target genes were significantly enriched in inflammation-related pathways such as Toll-like receptor signaling pathway, MAPK signaling pathway, and NOD-like receptor signaling pathway (Figure 2B and Table S4). Reactome analysis further supported that pathways such as PI5P, PP2A and IER3 Regulate PI3K/AKT Signaling, Activation of the AP-1 family of transcription factors, MAP kinase activation, and Interleukin-2 family signaling were core regulatory modules (Figure 2C), suggesting that these pathways may be potentially involved in the effects of EGCG. At the GO-BP level (Table S5), genes were significantly enriched in Response to oxygen-containing compound and Cellular response to oxygen-containing compound. At the GO-MF level (Table S6), enriched functions were highly concentrated in cytokine receptor binding, protein serine kinase activity, protein serine/threonine kinase activity, MAP kinase activity, protein kinase activity, kinase activity, and protein domain specific binding. At the GO-CC level (Table S7), gene products were significantly distributed in transcription factor AP-1 complex and protein-containing complex (Figure 2D). We further performed KEGG and GO enrichment analysis again using the DAVID database. KEGG pathway enrichment analysis showed (Figure 2E) that EGCG targets were significantly enriched in the cytosolic DNA-sensing pathway, the NOD-like receptor signaling pathway, the C-type lectin receptor signaling pathway, the toll-like receptor signaling pathway, the RIG-I-like receptor signaling pathway, the cytokine–cytokine receptor interaction, the FoxO signaling pathway, and the insulin signaling pathway. GO analysis results showed (Figure 2F) that EGCG targets were significantly enriched in negative regulation of inflammatory response, inflammatory response, immune response, innate immune response, cytokine activity, insulin receptor signaling pathway, and phosphatidylinositol 3-kinase/protein kinase B signal transduction. These results collectively suggest that EGCG may be associated with oxidative stress, ER stress, and inflammation, and that the ISR may be potentially involved. However, since these findings are based on enrichment analysis, further experimental validation is needed to confirm whether these pathways are functionally activated or modulated by EGCG.

### 3.3. Integrated Stress Response and Inflammation-Related Genes Are Also Potential Molecular Targets of Mn Toxicity

**Figure 3:**
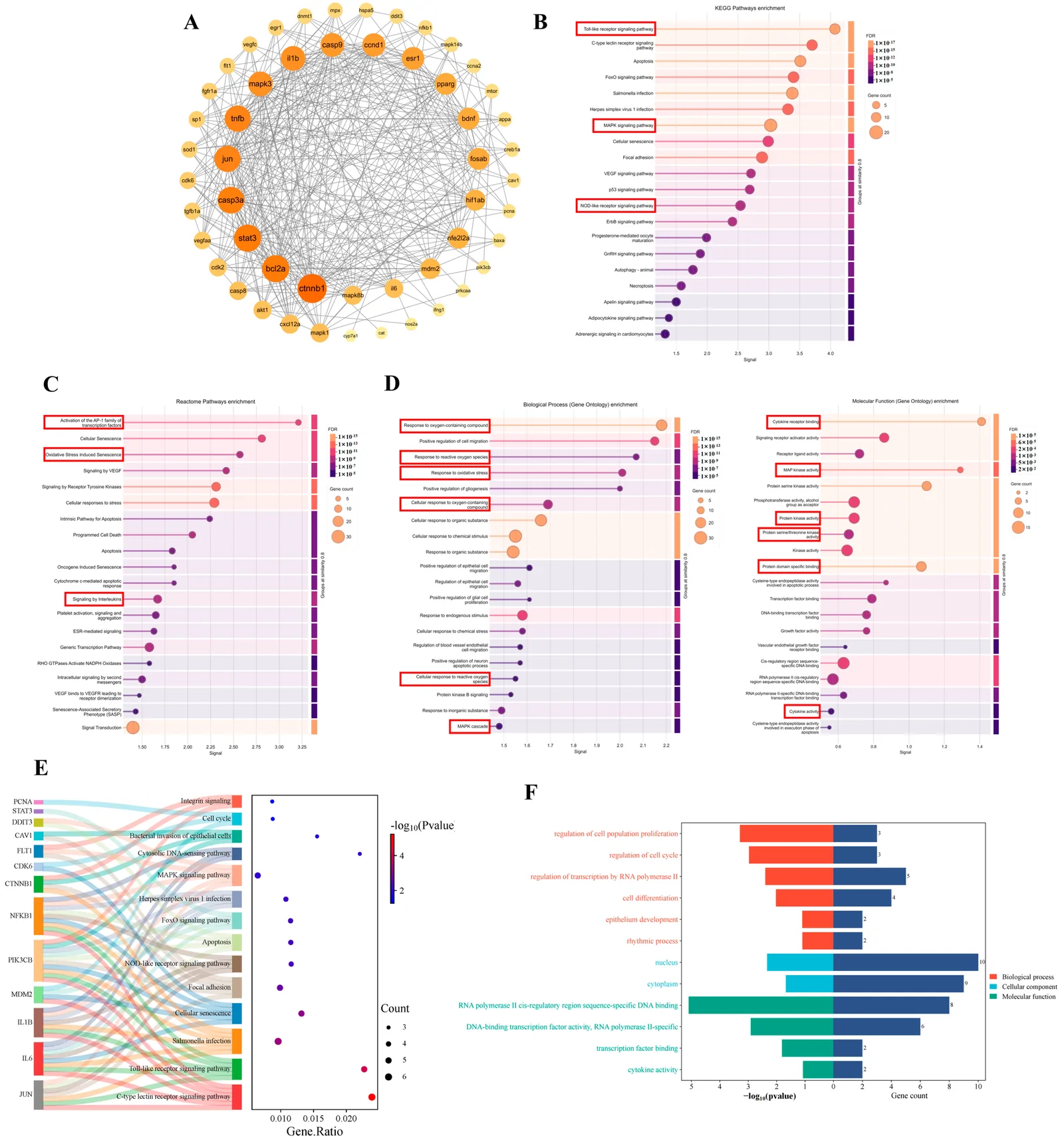
Integrated stress response and inflammation-related genes are also potential molecular targets of Mn toxicity. (**A**) Venn diagram showing the intersection of 3136 Mn toxicity targets from the CTD and 124 EGCG targets, yielding 59 common targets. (**B**) KEGG pathway enrichment analysis of the intersecting targets using the String database. (**C**) Reactome pathway enrichment analysis of the intersecting targets. (**D**) GO enrichment analysis of the intersecting targets using the String database. (**E**) KEGG pathway enrichment analysis of the intersecting targets using the DAVID database. (**F**) GO enrichment analysis of the intersecting targets using the DAVID database. The red boxes highlights KEGG pathways or GO terms related to oxidative stress, endoplasmic reticulum stress, and inflammation.

The CTD yielded 3136 potential toxicity targets of Mn (Table S8). By intersecting the potential Mn toxicity targets with the potential regulatory targets of EGCG, we obtained 59 potential EGCG targets for alleviating Mn toxicity (Table S9). Among these, inflammation-related genes (*NFKB1*, *TNF*, *IL6*, *IL1B*, *IFNG*, *MAPK8*, etc.), oxidative stress-related genes (*NFE2L2*, *SOD1*, *CAT*, *HIF1A*, *FOXO3*, *NOS2*, *DDIT3*, etc.), and ER stress-related genes (*HSPA5*, *DDIT3*, etc.) occupied significant positions (Figure 3A). KEGG pathway enrichment analysis using the String database showed that the target genes were significantly enriched in inflammation-related pathways such as the Toll-like receptor signaling pathway, the MAPK signaling pathway, and the NOD-like receptor signaling pathway (Figure 3B and Table S10). Reactome analysis identified significantly enriched inflammation-related pathways such as Activation of the AP-1 family of transcription factors and Signaling by Interleukins, as well as the oxidative stress-related pathway Oxidative Stress-Induced Senescence (Figure 3C). At the GO-BP level (Table S11), genes were significantly enriched in response to oxygen-containing compound, response to reactive oxygen species, response to oxidative stress, cellular response to oxygen-containing compound, cellular response to reactive oxygen species, and MAPK cascade. At the GO-MF level (Table S12), enriched functions were highly concentrated in cytokine receptor binding, MAP kinase activity, protein kinase activity, protein serine/threonine kinase activity, protein domain specific binding, and cytokine activity (Figure 3D). We further performed KEGG and GO enrichment analysis again using the DAVID database. KEGG pathway enrichment analysis showed that EGCG targets were significantly enriched in the NOD-like receptor signaling pathway, the toll-like receptor signaling pathway, the C-type lectin receptor signaling pathway, the Cytosolic DNA-sensing pathway, and the FoxO signaling pathway (Figure 3E). GO analysis results showed that the intersecting targets of EGCG and Mn were significantly enriched in terms such as cytokine activity (Figure 3F). These enrichment analysis results suggest that the mechanism by which EGCG alleviates Mn toxicity may involve oxidative stress, ER stress, and inflammation.

### 3.4. EGCG Attenuated Mn-Associated Oxidative Stress and Stress-Related Transcript Changes

**Figure 4:**
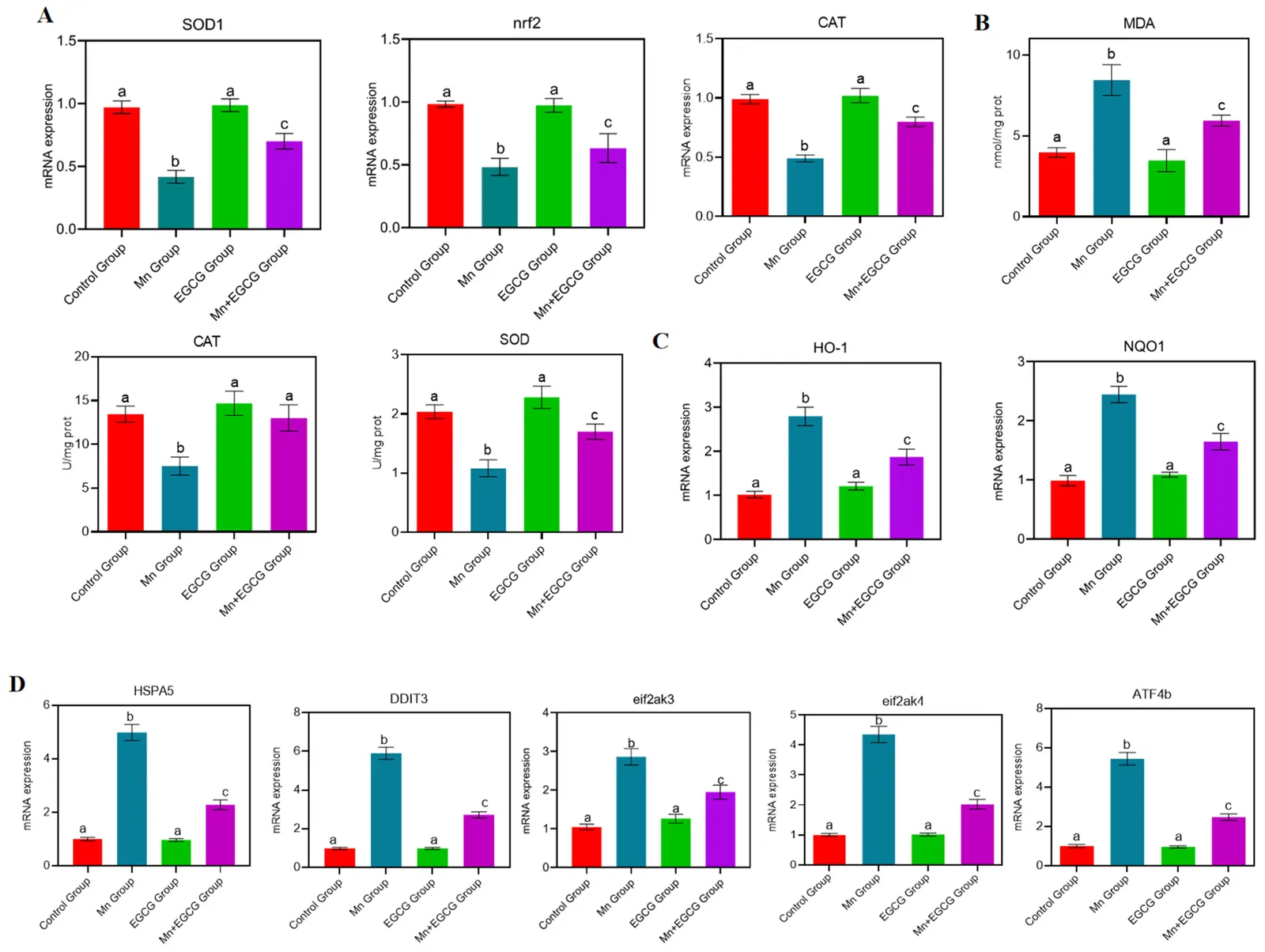
EGCG attenuated Mn-associated oxidative stress and stress-related transcript changes. (**A**) Relative mRNA expression levels of the antioxidant genes *SOD1*, *NRF2*, and *CAT* in carp heart tissues. (**B**) Activities of SOD and CAT, and MDA content in carp heart tissues. (**C**) Relative mRNA expression levels of *HO-1* and *NQO1*. (**D**) Relative mRNA expression levels of ER stress and the ISR marker genes *HSPA5*, *DDIT3*, *eif2ak3*, *eif2ak4*, and *atf4b* in carp heart tissues. Data are expressed as mean ± SD (*n* = 3 tanks per group). Different lowercase letters indicate significant differences between groups (*p* < 0.05).

Based on the database analysis above, we hypothesized that the ISR is an important mechanism by which EGCG alleviates Mn toxicity, primarily involving oxidative stress and ER stress signaling. Therefore, we measured the abundance of selected transcripts and biochemical indices to assess treatment-associated changes. We found that Mn exposure decreased the mRNA expression levels of *SOD1*, *NRF2*, and *CAT*, which was reversed by EGCG supplementation (Figure 4A). Consistent results were obtained for the SOD and CAT activity assays (Figure 4B), as well as *HO-1* and *NQO1* mRNA expressions (Figure 4C), indicating that Mn exposure suppressed the cardiac antioxidant defense system, while EGCG positively regulated the antioxidant system. MDA content measurement showed that EGCG alleviated the Mn-induced increase in MDA content, indicating that EGCG supplementation effectively alleviated Mn-induced oxidative stress.

*HSPA5*, *DDIT3*, *eif2ak4* (encoding GCN2), and *atf4b* are markers of ER stress and core components of the ISR. As shown in Figure 4D, compared with the control and EGCG groups, the mRNA expression levels of *HSPA5*, *DDIT3*, *eif2ak4*, and *atf4b* in the carp heart tissues of the Mn-treated group were increased, suggesting that Mn exposure may induce ER stress and ISR-related transcript changes in cardiac tissue. Compared with the Mn group, the mRNA expression of *HSPA5*, *DDIT3*, *eif2ak4*, and *atf4b* in the Mn+EGCG combined treatment group was decreased, suggesting that EGCG supplementation may attenuate Mn-induced ER stress and ISR-related gene expression in common carp hearts, mitigating ER stress-mediated cellular damage.

### 3.5. EGCG Alleviates Mn-Induced Inflammatory Responses

**Figure 5:**
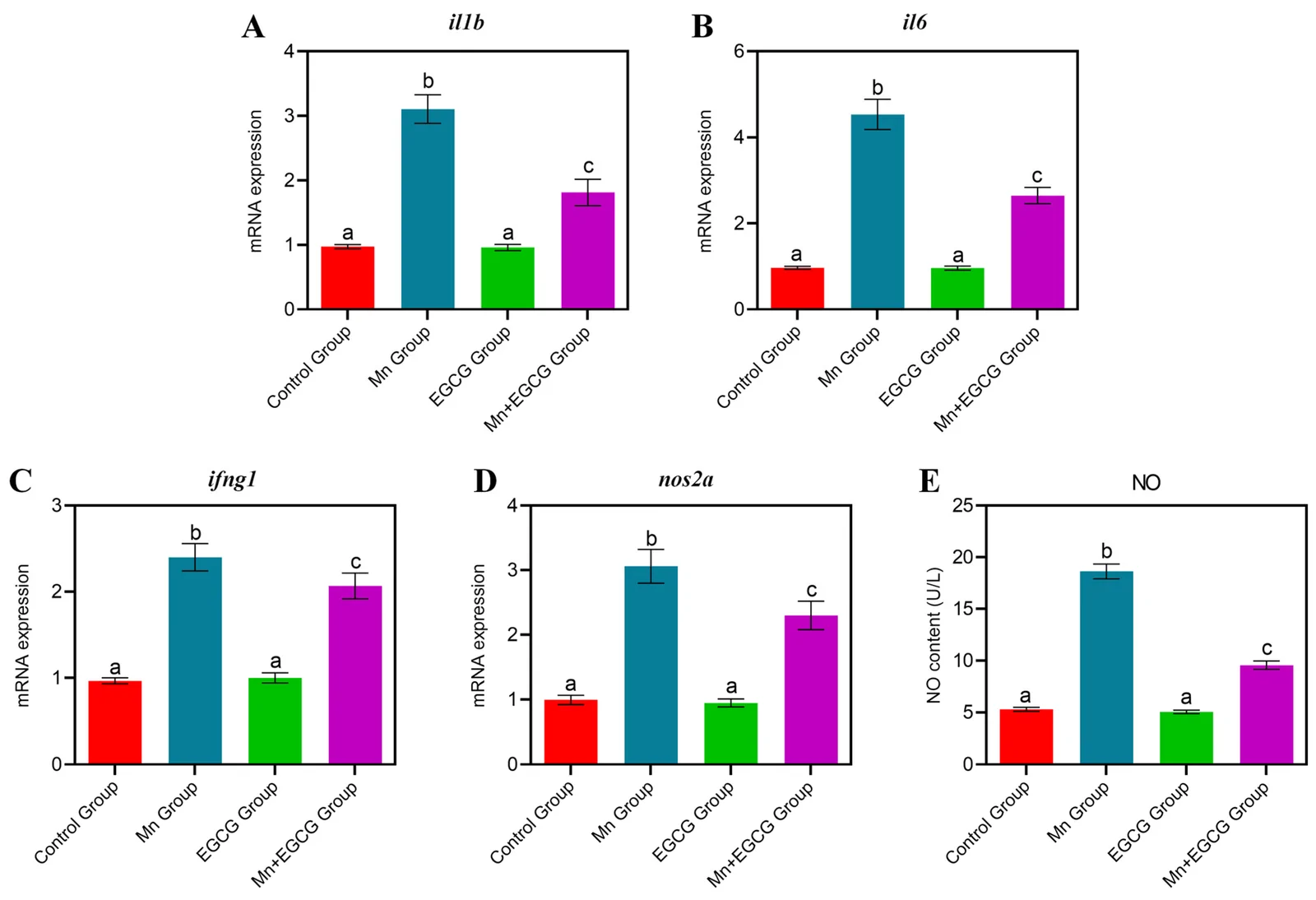
EGCG alleviated Mn-induced inflammatory responses. Relative mRNA expression levels of pro-inflammatory cytokines (**A**) *il1b*, (**B**) *il6*, (**C**) *ifng1*, (**D**) *nos2a*, and (**E**) NO content in carp heart tissues. Data are expressed as mean ± SD (*n* = 3 tanks per group). Different lowercase letters indicate significant differences between groups (*p* < 0.05).

Since inflammation-related genes were common targets of EGCG and Mn, and inflammation can be a downstream biological event of the ISR, we measured inflammatory cytokines. Compared with the control group, the mRNA expression levels of the pro-inflammatory cytokines *il1b* (Figure 5A), *il6* (Figure 5B), *ifng1* (Figure 5C), and *nos2a* (Figure 5D) and NO content (Figure 5E) in the carp heart tissues of the Mn-treated group were increased, indicating that Mn exposure induced an inflammatory response in cardiac tissue. There were no significant differences in the above indicators between the EGCG-alone group and the control group, indicating that EGCG alone had no significant effect on the expression of cardiac inflammation-related factors. Compared with the Mn group, the mRNA expression levels of *il1b*, *il6*, *ifng1*, and *nos2a* and NO content in the Mn+EGCG combined treatment group were decreased, but still higher than the control group levels. These results indicate that EGCG supplementation effectively inhibited Mn-induced cardiac inflammation in carp.

### 3.6. EGCG Attenuated Mn-Associated Changes in Inflammatory Signaling-Related Transcripts

**Figure 6:**
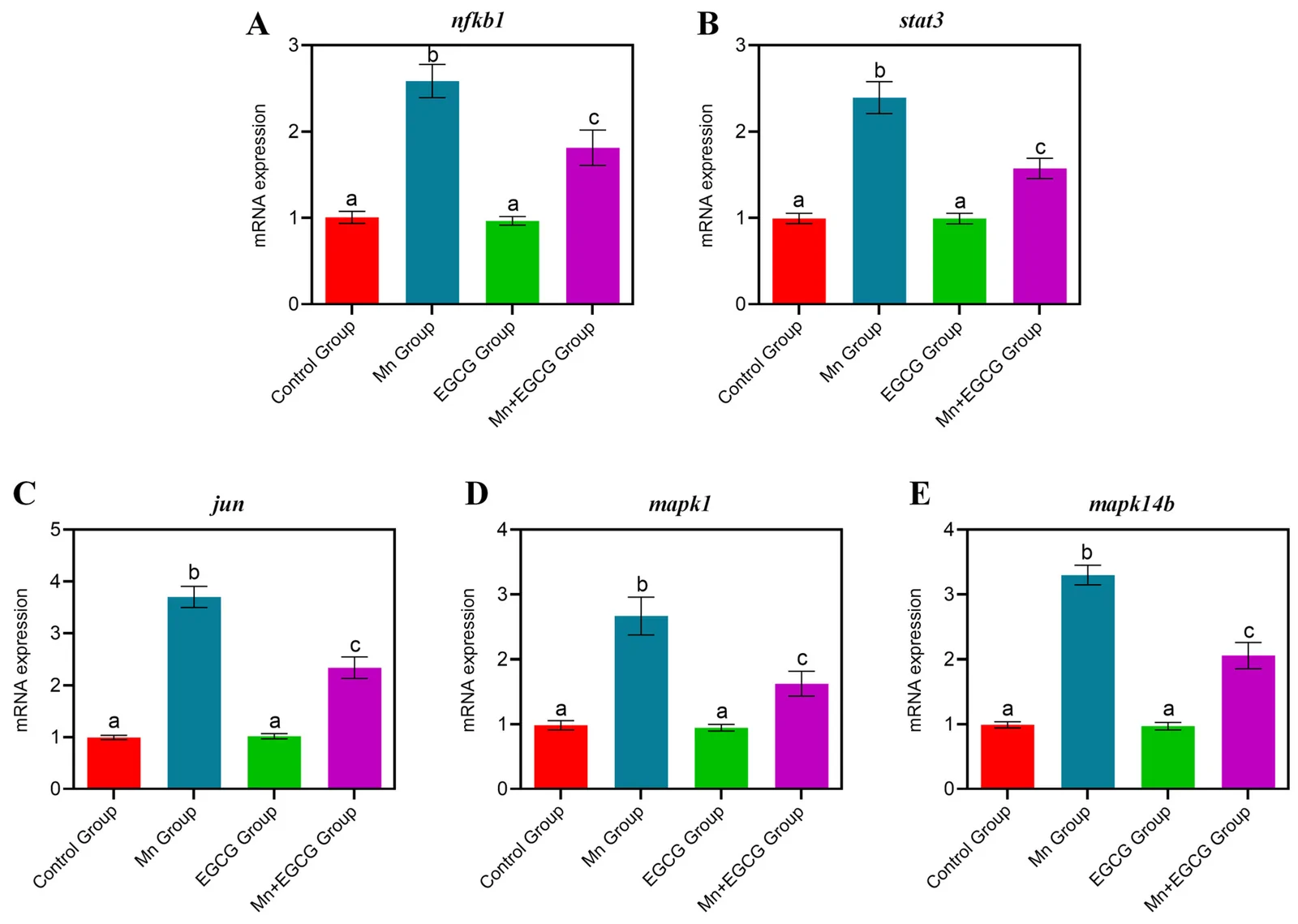
EGCG attenuated Mn-associated changes in inflammatory signaling-related transcripts. Relative mRNA expression levels of the inflammatory signaling pathway key nodes (**A**) *nfkb1*, (**B**) *stat3*, (**C**) *jun*, (**D**) *mapk1*, and (**E**) *mapk14b* in carp heart tissues. Data are expressed as mean ± SD (*n* = 3 tanks per group). Different lowercase letters indicate significant differences between groups (*p* < 0.05).

Inflammation is primarily driven by core molecules in pro-inflammatory signaling pathways. *nfkb1*, *stat3*, *jun*, *mapk1*, and *mapk14b* are key nodes in inflammatory signaling pathways. Interestingly, these genes were also predicted as potential molecular targets of Mn and EGCG. Therefore, these signaling molecules may represent a potential mechanism linking the ISR to inflammation in the context of EGCG alleviating Mn toxicity. We also detected *nfkb1* (Figure 6A), *stat3* (Figure 6B), *jun* (Figure 6C), *mapk1* (Figure 6D), and *mapk14b* (Figure 6E). Compared with the control group, the mRNA expression levels of *nfkb1*, *stat3*, *jun*, *mapk1*, and *mapk14b* in the carp heart tissues of the Mn-treated group were increased, indicating that Mn exposure altered the expression of genes associated with the NF-κB, STAT3, and MAPK pathways in cardiac tissue, and these transcriptional changes were accompanied by Mn-induced inflammatory responses. There were no significant differences in the expression of these genes between the EGCG-alone group and the control group, indicating that EGCG alone had no significant effect on the expression of key genes in inflammatory signaling pathways. Compared with the Mn group, the mRNA expression of *nfkb1*, *stat3*, *mapk1*, *mapk14b*, and *jun* in the Mn+EGCG combined treatment group was decreased, suggesting that EGCG supplementation may reverse the Mn-induced upregulation of key genes involved in inflammatory signaling pathways in carp hearts, which may be one of the important molecular mechanisms underlying the anti-inflammatory effects of EGCG.

### 3.7. ISR-Associated Signaling Is Involved in the Mn-Induced Inflammatory Response

**Figure 7:**
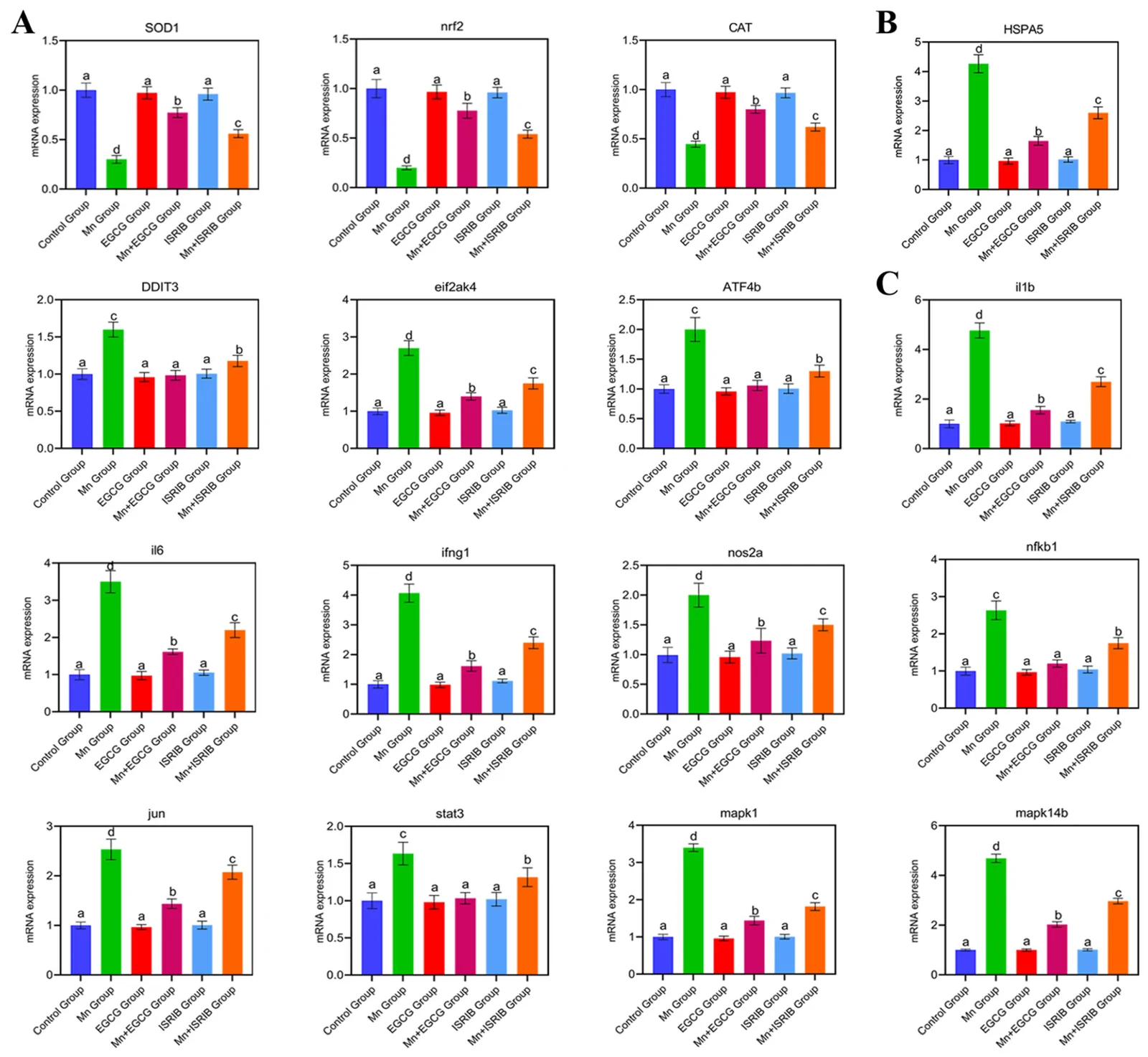
ISR-associated signaling is involved in the Mn-induced inflammatory response. EPC cells were treated with Mn (150 µM), EGCG (250 µM), and/or ISRIB (200 nM) for 24 h. (**A**) Relative mRNA expression levels of the antioxidant genes *SOD1*, *NRF2*, *CAT*, *HO-1*, and *NQO1*. (**B**) Relative mRNA expression levels of ER stress and the ISR genes *HSPA5*, *DDIT3*, *eif2ak4*, and *atf4b*. (**C**) Relative mRNA expression levels of inflammation-related genes (*il1b*, *il6*, *ifng1*, *nos2a*) and inflammatory pathway key molecules (*nfkb1*, *stat3*, *mapk1*, *mapk14b*, *jun*). Data are expressed as mean ± SD (*n* = 3). Different lowercase letters indicate significant differences between groups (*p* < 0.05).

**Figure 8:**
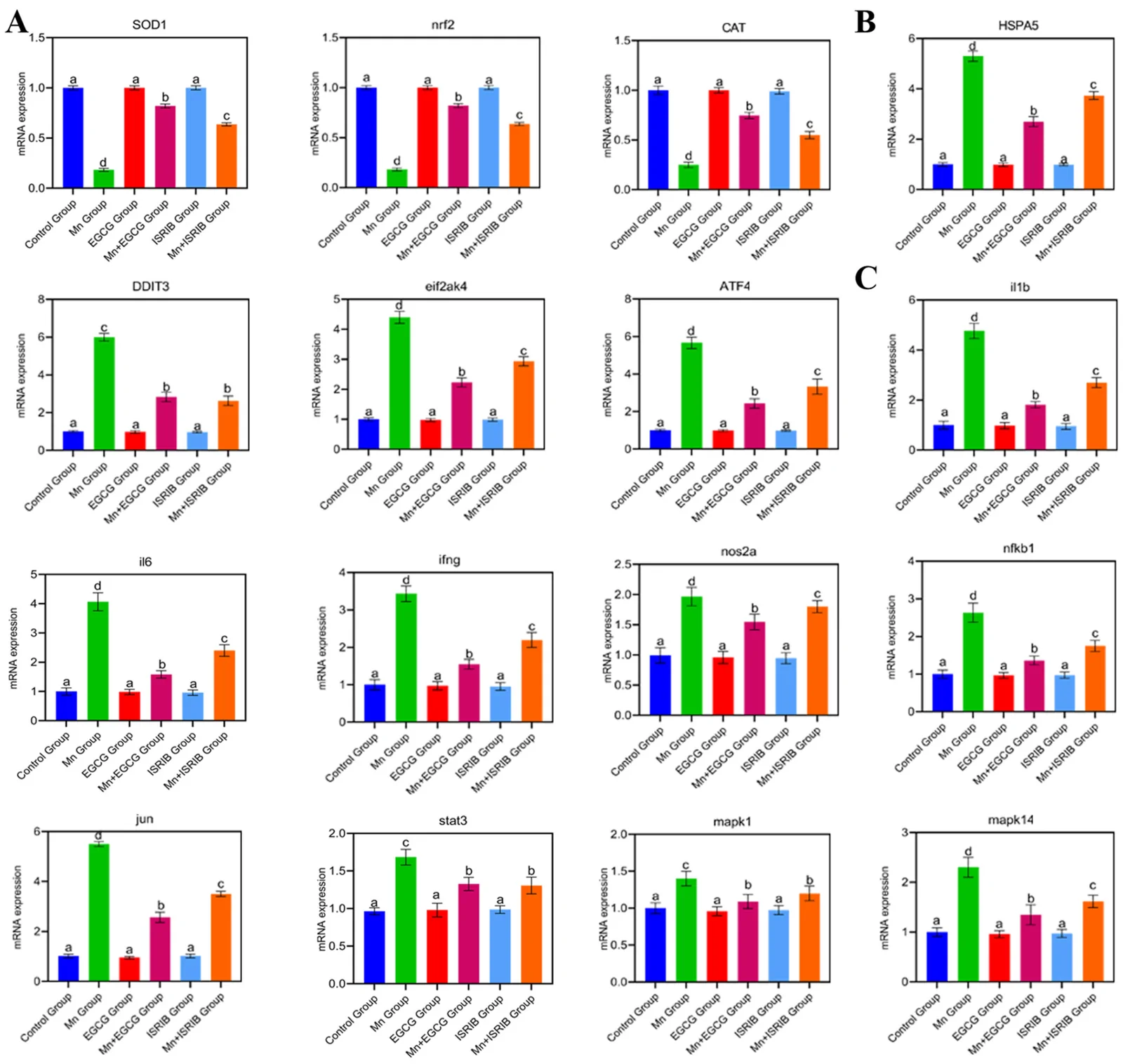
Effects of EGCG and ISRIB on Mn-associated transcript changes in H9c2 cells (200 µM), EGCG (20 µM), and/or ISRIB (200 nM) for 24 h. (**A**) Relative mRNA expression levels of the antioxidant genes *SOD1*, *NRF2*, *CAT*, *HO-1*, and *NQO1*. (**B**) Relative mRNA expression levels of ER stress and the ISR genes *HSPA5*, *DDIT3*, *eif2ak4*, and *atf4*. (**C**) Relative mRNA expression levels of inflammation-related genes (*il1b*, *il6*, *ifng1*, *nos2a*) and inflammatory pathway key molecules (*nfkb1*, *stat3*, *mapk1*, *mapk14*, *jun*). Data are expressed as mean ± SD (*n* = 3). Different lowercase letters indicate significant differences between groups (*p* < 0.05).

To investigate the potential involvement of ISR-associated signaling in the inflammatory response induced by Mn, we performed in vitro mechanistic validation using carp epithelioma papulosum (EPC) cells (Figure 7A–C) and rat cardiomyocytes (H9c2 cells) (Figure 8A–C). Cells exposed to Mn were co-treated with ISRIB, a pharmacological modulator of ISR signaling that enhances eIF2B activity and counteracts the translational effects associated with eIF2α phosphorylation. The expression of ISR-associated genes and representative inflammatory cytokines was subsequently examined to assess whether modulation of ISR-associated signaling was accompanied by changes in the Mn-induced inflammatory response.

In EPC cells, Mn decreased the mRNA expression of the antioxidant genes *SOD1*, *NRF2*, *CAT*, *HO-1*, and *NQO1*, which was alleviated by ISRIB supplementation (Figure 7A). ISRIB also reversed the Mn-induced upregulation of ER stress and the ISR genes *HSPA5*, *DDIT3*, *eif2ak4*, and *atf4b* (Figure 7B), suggesting that ISRIB attenuated Mn-induced ISR-related gene expression. Moreover, Mn exposure significantly increased the expression of inflammation-related genes (*il1b*, *il6*, *ifng1*, and *nos2a*) and key inflammatory signaling molecules (*nfkb1*, *stat3*, *mapk1*, *mapk14b*, and *jun*), whereas these transcriptional responses were attenuated by ISRIB treatment (Figure 7C). In H9c2 rat cardiomyocytes, we performed the same detections and obtained consistent conclusions with EPC cells for antioxidant genes (Figure 8A), ER stress and the ISR genes (Figure 8B), and inflammatory genes (Figure 8C). Moreover, we determined protein levels of p-eIF2α, eIF2α, ATF4, CHOP, PERK, GCN2, NF-κB, STAT3, MAPK1, MAPK14B, and JUN, as well as calculated p-eIF2α/eIF2α in H9c2 cells (Figure S2). We found that, except eIF2α, all the factors were upregulated under Mn exposure, and they were reversed by EGCG or ISRIB supplement. These observations are consistent with ISR-related and inflammatory transcript changes induced by Mn in two different cell models; moreover, through ISR-specific inhibition experiments, our data suggest that ISR-related signaling may contribute to Mn-induced inflammatory responses, as ISR inhibition significantly attenuated the upregulation of inflammatory genes. However, we do not identify the responsible upstream kinase, establish the direction of causality, or demonstrate ATF4 promoter binding and direct regulation of inflammatory genes.

## 4. Discussion

To avoid overinterpretation, we use the term “cardiotoxicity” only as a broad research concept. The experimental evidence obtained here supports Mn-induced cardiac injury, biochemical cardiac stress, and myocardial structural/histopathological damage, but not functional cardiac impairment. This study found that Mn was associated with myocardial structural injury, altered serum enzyme activities, impaired antioxidant indices and changes in stress-associated and inflammatory transcripts. Concurrent dietary EGCG supplementation attenuated these changes. Together with the ISRIB cell experiments, these observations support investigation of a possible association between ISR-related responses and inflammation. These findings reveal a potential link among Mn-induced cellular stress, ISR-associated signaling, inflammatory responses, and cardiac injury, providing a framework for further investigating the molecular mechanisms underlying heavy metal-induced cardiac injury, and support further evaluation of EGCG as an experimental anti-stress dietary intervention.

### 4.1. ISR: The Signaling Hub Connecting Oxidative Stress and ER Stress

In this study, Mn exposure led to decreased expression and activity of antioxidant enzyme genes (*SOD1*, *CAT*, *NRF2*) and increased MDA content in carp heart tissue, consistent with the occurrence of oxidative stress. Simultaneously, mRNA expression of ER stress marker genes *HSPA5* and *DDIT3*, as well as the ISR core components *eif2ak4* and *atf4b*, were significantly upregulated, suggesting concurrent ER stress and ISR-related changes. This result is consistent with findings from other researchers. Mn exposure has been shown to induce oxidative stress and ER stress, specifically manifested as significantly elevated levels of the lipid peroxidation product MDA [18]. In cell models, Mn exposure can also alter the expression of the ER stress-related protein GRP78 (encoded by *HSPA5*) [19], accompanied by decreased activities of antioxidant enzymes SOD and CAT [18]. These findings indicate that Mn induces oxidative damage and ER stress by elevating stress indicators like MDA and *HSPA5* while reducing antioxidant enzyme activities such as SOD and CAT. It should be emphasized that the in vivo evidence from common carp heart tissue in this study is limited to histopathological, biochemical, and transcript-level analyses. No protein-level measurements of ISR activation were performed in carp heart tissue. Therefore, the present data do not directly confirm ISR activation in the carp heart. The protein-level ELISA data were obtained only in H9c2 rat cardiac-derived myoblasts. The ISRIB experiments in EPC and H9c2 cells provide pharmacological evidence that ISR-associated signaling may contribute to Mn-induced inflammatory gene expression in cell models, but they do not establish direct ISR activation in carp heart or a causal ISR–inflammation axis in vivo.

The ISR can play a central role in Mn cardiotoxicity due to its unique signal integration mechanism. Four classes of ISR upstream kinases, PERK (sensing ER stress), PKR (sensing oxidative stress and viral infection), GCN2 (sensing amino acid deprivation), and HRI (sensing heme deficiency), can all phosphorylate the common substrate eIF2α, although eIF2α may not necessarily be the same, converging diverse cellular damage signals onto a single molecular node [20,21]. This multiple-input and single-node architecture enables the ISR to convert stressors of vastly different natures into a unified transcriptional–translational regulatory response. Studies have shown that mammalian cells activate the ISR to effectively cope with various stress conditions and maintain normal function, and the ISR achieves this through phosphorylation of eIF2α (Ser51) by the four kinases PERK, PKR, GCN2, and HRI to regulate ISR signaling and intracellular protein translation [22]. Manganese, as a heavy metal capable of inducing both oxidative stress and ER stress, has been shown to primarily induce oxidative stress by promoting excessive production of reactive oxygen species (ROS) [23], and can inhibit antioxidant gene expression through epigenetic regulation, leading to mitochondrial oxidative damage [24]. Meanwhile, Mn exposure has also been confirmed to directly trigger ER stress responses, activating signaling pathways such as PERK-eIF2α and IRE1 and upregulating the expression of stress marker proteins like GRP78 and CHOP [25,26]. Its toxic effects overlap with the upstream activation pathways of the ISR; therefore, it is plausible that the ISR is induced in Mn-caused cardiac injury. Its toxic effects precisely cover the two main activation pathways of the ISR; therefore, it is logical that ISR-related signaling is engaged in Mn-caused cardiac injury. This study further investigate the relationship between ISR-associated signaling and inflammation through intervention experiments with the ISR-specific inhibitor ISRIB in EPC cells and H9c2 cells: ISRIB not only reversed Mn-induced downregulation of antioxidant genes and upregulation of ER stress and the ISR genes, but also concurrently alleviated the expression of inflammatory factors, thereby providing pharmacological evidence supporting the potential involvement of ISR-associated signaling in the Mn-induced inflammatory response, although these data alone do not establish a direct upstream causal relationship between ISR and inflammation.

### 4.2. Association Between ISR-Related Responses and Inflammation

Inflammation is another key pathological pathway revealed in this study. Mn exposure significantly upregulated the mRNA expression of pro-inflammatory cytokines *il1b*, *il6*, *ifng1*, *nos2a* and NO content in carp hearts, while the transcriptional levels of the core nodes of inflammatory signaling pathways, including *nfkb1*, *stat3*, *jun*, *mapk1b*, and *mapk14b*, were also simultaneously elevated. These results are consistent with the findings of Zhou et al. (2023) in Mn-exposed carp kidneys, indicating that inflammation is a common pathological event of Mn toxicity across multiple organs in aquatic animals [14]. Additional studies corroborate this: in marine medaka embryos, Mn exposure significantly upregulated the expression of TNFα and il1β [27]; in largemouth bass liver, Mn exposure induced upregulation of pro-inflammatory factors such as IL-1β and TNF-α [28]; in grass carp gill tissues, both manganese deficiency and excess could induce inflammatory responses via activation of the NF-κB p65 signaling pathway [29]. Furthermore, a review by Cheng et al. (2023) further pointed out at the molecular level that the NF-κB and JAK-STAT signaling pathways are core regulatory nodes in manganese-induced neuroinflammation [30]. The above evidence indicates that inflammation is a common pathological event of Mn toxicity across multiple organs in aquatic animals. However, the core breakthrough of this study lies in observing association between ISR-related signaling and the inflammatory response. In EPC cells and H9c2 cells, inhibition of the ISR with ISRIB significantly alleviated Mn-induced upregulation of inflammatory cytokines, demonstrating that ISR-associated signaling may contribute to the Mn-induced inflammatory response rather than simply occurring independently of inflammation. This finding is highly consistent with recent cutting-edge advances in the field of mammalian immunology. Asada et al. (2025) found in tissue-resident memory CD4^+^ T cells that the ISR-eIF2α pathway maintains immune quiescence under steady-state conditions by inhibiting the translation of pro-inflammatory cytokine mRNAs, and genetic or pharmacological activation of this pathway can reduce cytokine production and alleviate autoimmune kidney disease [31]. Wang et al. (2025) confirmed in a type 1 diabetes model that the ISR is a key regulator of macrophage-driven inflammation and migration [32]. In this study, the similar responses observed in EPC and H9c2 cells provide evidence consistent with an association between ISR-related signaling and inflammation. However, it is worth noting that Our evidence is limited to common carp under the tested exposure conditions and the two cell models. Confirmation in primary carp cardiomyocytes and additional species is needed before broader generalization.

Regarding the specific molecular mechanisms by which the ISR influences inflammation, this study provides preliminary clues. Mn exposure simultaneously upregulated the expression of inflammatory signaling core nodes including *nfkb1*, *stat3*, *mapk1*, *mapk14b*, and *jun*, and these genes were also predicted as common targets of Mn toxicity and EGCG in bioinformatics analysis. It should be noted that in this study, nfkb1 (encoding p50) was examined at the transcript level in carp heart tissues, whereas p65 (encoded by RELA) is mentioned only in the context of the cited literature; therefore, the present transcript data do not directly address RELA/p65-mediated signaling. The NFKB1 gene encodes the p50 protein of the NF-κB transcription factor family, initiating the transcription of hundreds of pro-inflammatory cytokines and chemokines such as IL6, TNF, and IL8 [33]. Its sustained activation in chronic inflammation promotes the expression of inflammatory mediators and anti-apoptotic genes, creating a favorable microenvironment for tumor development [33]. STAT3 is a key transcription factor in the JAK-STAT pathway; in inflammation, STAT3 can act as a signaling hub that promotes inflammation (e.g., mediating IL-6-driven chronic inflammation) [34]. MAPK1 (i.e., ERK2) belongs to the MAPK family and transmits extracellular growth and stress signals through the classical Ras-Raf-MEK-ERK cascade. In inflammation, activation of MAPK1 can upregulate the expression of pro-inflammatory chemokines, promoting the formation of an inflammatory microenvironment [35]. MAPK14 is the most extensively studied isoform of the p38 MAPK family and the most widely expressed kinase in inflammatory cells. It is a core regulator of pro-inflammatory cytokine synthesis, with major mechanisms including: increasing translation efficiency by stabilizing ARE-containing mRNAs (e.g., TNF, IL6), and enhancing transcription of pro-inflammatory genes by phosphorylating transcription factors such as ATF-2 [36]. Based on its critical pro-inflammatory function, its inhibitors have shown significant efficacy in various preclinical disease models, including rheumatoid arthritis and inflammatory bowel disease, making it an important target for anti-inflammatory drug development [36]. JUN is a core component of the AP-1 transcription factor complex [37]. In inflammation, c-Jun/AP-1 serves as a critical switch connecting external stimuli to inflammatory gene expression, directly regulating the transcription of various inflammatory mediators. We hypothesize that in Mn-exposed cardiomyocytes, the ISR influences the transcriptional activation of pro-inflammatory factors through interactions with the NF-κB, STAT3, JUN, and MAPK signaling pathways. This hypothesis requires further verification in future studies using techniques such as chromatin immunoprecipitation and dual-luciferase reporter assays.

### 4.3. EGCG-Associated Attenuation of Cellular Stress and Inflammatory Responses

EGCG demonstrated significant cardioprotective effects in this study. Dietary EGCG supplementation not only reversed Mn-induced histopathological changes, including disorganized and broken myocardial fibers and inflammatory cell infiltration, but also restored serum myocardial injury markers (AST, LDH, CK, α-HBDH) and myocardial ATPase activities. It should be noted that AST and LDH are not heart-specific enzymes in fish, as they can also be released from liver and skeletal muscle. In the present study, these markers were used as auxiliary indicators and interpreted together with cardiac histopathological damage to support the occurrence of myocardial injury.

At the molecular level, EGCG simultaneously inhibited oxidative stress (restoring *SOD1*, *CAT*, *NRF2* expression, reducing MDA), ER stress and the ISR (reducing *HSPA5*, *DDIT3*, *eif2ak4*, *atf4b*), and inflammatory responses (reducing *il1b*, *il6*, *ifng1*, *nos2a*, as well as *nfkb1*, *stat3*, *mapk1*, *mapk14b*, and *jun*), displaying a multi-target and multi-pathway regulatory profile.

EGCG attenuates oxidative stress and ER stress-associated responses, thereby reducing upstream stress inputs and secondarily changing ISR-associated and inflammatory responses. In cancer research, EGCG can induce ER stress by activating the PERK-eIF2α-ATF4 pathway, thereby promoting tumor cell apoptosis. Md Nesran et al. (2019) confirmed in colorectal cancer cells that EGCG could upregulate the expression of PERK, phosphorylated eIF2α, and ATF4, and induce apoptosis through activation of Caspase 3/7 [38]. Furthermore, Wang et al. (2017) found that EGCG could enhance ER stress-induced cancer cell apoptosis by inhibiting PARP16 activity, with mechanisms involving inhibition of PERK phosphorylation and transcription of ER stress-related genes [39]. However, under the conditions of this study, EGCG acted to inhibit Mn-increased ISR-related factors. As the most abundant polyphenolic active component in green tea, EGCG has been confirmed by multiple studies to exert significant cytoprotective effects in various disease models and stress conditions by targeting and inhibiting key signaling molecules of ER stress. In glioblastoma research, Pyrko et al. found that EGCG could target the ATP-binding domain of GRP78 (encoded by *HSPA5*) and block its anti-apoptotic function, thereby relieving its inhibition of the pro-apoptotic factor CHOP (encoded by *DDIT3*) and enhancing chemosensitivity to temozolomide [40]. In trabecular meshwork cells, Zhou et al. demonstrated that EGCG pretreatment significantly inhibited tunicamycin-induced upregulation of *HSPA5*, *DDIT3*, and *ATF4* mRNA expression, thereby protecting cells from ER stress-mediated cell death [41]. In a lead-exposed rat model of hepatic insulin resistance and apoptosis, Liu et al. found that A-type dimeric EGCG significantly reduced GRP78 expression levels and inhibited the activation of PERK (encoded by EIF2AK3), ATF4, and CHOP, thereby alleviating ER stress and improving insulin sensitivity [42]. In summary, EGCG exerts broad-spectrum cytoprotective effects by modulating the expression and function of *HSPA5*/GRP78 through multiple targets and inhibiting the cascade activation of the PERK-ATF4-CHOP signaling axis, effectively alleviating ER stress under various pathological conditions including glioma, heavy metal toxicity, and metabolic disorders. This seemingly contradictory bidirectional regulation precisely reflects the nature of the ISR as a cellular stress sensor: under mild stress, the ISR helps cells adapt and survive; under excessive or irreversible stress, the ISR shifts toward promoting cell death. However, these studies provide context for interpreting our observations, but do not establish a universal mode of EGCG action or direct ISR inhibition in carp. Moreover, in this study, the observed changes in *atf4b*- and inflammation-related gene expression do not demonstrate direct regulation of NF-κB-, STAT3-, or MAPK-associated responses by ATF4. Thus, we can but hypothesize that the role of EGCG is not simply to activate or inhibit the ISR, but rather to restore Mn-induced ISR-related changes toward homeostatic levels to a homeostatic level, which may be one of the core mechanisms underlying its broad pharmacological activities.

### 4.4. Limitations of the Study

This study has several limitations. First, the animal experiment used only a single Mn exposure concentration (3.5 mg/L Mn^2+^) and a single EGCG dose (75 mg/kg diet), without examining the dose–response relationship, threshold effects, the optimal EGCG concentration, or the therapeutic window. Moreover, the Mn exposure protocol involved a single metal exposure, which differs from the combined pollution scenarios of multiple heavy metals coexisting in actual aquatic environments. Second, this study did not track the dynamic evolution of the ISR from adaptive protection to pathological injury. Third, although the intervention with ISRIB attenuated Mn-induced transcriptional changes in ISR-associated and inflammatory genes, these findings alone are insufficient to establish that ISR is directly upstream of inflammation. Therefore, the present findings support the possibility that ISR-associated signaling may contribute to the Mn-induced inflammatory response, rather than establishing a direct causal relationship. In addition, whether the ISR downstream transcription factor ATF4 directly binds to the promoters of inflammatory signaling genes (nfkb1, stat3, mapk1, etc.) and drives their transcription requires further verification through experiments such as ChIP-qPCR and dual-luciferase reporter assays. Fourth, tissue Mn concentrations and direct cardiac function were not measured. The lack of Mn quantification in cardiac tissue, liver, gill, or other tissues prevents us from verifying the specific manganese accumulation characteristics of cardiac, and the biochemical and histopathological indicators used here cannot directly demonstrate impaired systolic or diastolic function. Therefore, our conclusions are restricted to Mn-induced cardiac injury at the structural, biochemical, and molecular levels. Future studies should determine Mn concentrations in relevant tissues, particularly the heart, liver, and gill, to characterize tissue-specific Mn accumulation and further clarify the relationship between internal Mn exposure and toxicity. Fifth, EPC cells are epithelial cells rather than cardiomyocytes, and H9c2 cells are rat cardiac-derived myoblasts that do not represent primary carp cardiomyocytes. Their responses provide supporting mechanistic evidence, rather than direct confirmation of the proposed mechanism in carp cardiomyocytes. The study of one fish species and two cell models also does not establish a universal cross-species mechanism. Validation in primary carp cardiomyocytes and additional species is required. In addition, the protein-level validation by ELISA was performed in the H9c2 rat cardiac-derived cell model rather than in carp heart tissue, so protein-level evidence for ISR involvement in the carp heart in vivo is still lacking. Sixth, the histopathological assessment was semi-quantitative, based on eight parameters scored from 0 to 3. This provides an ordinal grading of cardiac lesions but does not provide continuous morphometric measurements, such as fibrotic area, inflammatory cell density, or cardiomyocyte cross-sectional area. And the semi-quantitative H&E scoring itself cannot capture subcellular changes. Seventh, although Tukey’s post-hoc test was used to control the family-wise error rate within each endpoint, no formal correction for multiple comparisons was applied across the different endpoints, which should be considered when interpreting the results. Eighth, although water temperature, pH and dissolved oxygen were maintained and monitored throughout the exposure, other water chemistry parameters that may influence Mn bioavailability (e.g., alkalinity and dissolved organic carbon) were not measured, which should be addressed in future studies. Ninth, β-actin was used as the sole reference gene for qRT-PCR normalization, and its stability was not systematically evaluated together with additional candidate reference genes under Mn and EGCG treatments. Future studies should validate multiple candidate reference genes under the specific experimental conditions and employ multiple stable reference genes where appropriate to improve the robustness of qRT-PCR normalization. In addition, future studies should perform a priori power analysis based on the number of tanks, not the number of individual fish, and should increase tank-level replication. Furthermore, water chemistry parameters, including alkalinity, and metal speciation, were not comprehensively monitored, which may have introduced additional uncertainty into the interpretation of the exposure effects. The histopathological evaluation was semi-quantitative rather than fully quantitative. Finally, the stability, bioavailability, pharmacokinetic characteristics of EGCG in feed, and its potential effects on fish growth performance all require systematic evaluation. These limitations suggest that future research should be further deepened in terms of dose–response relationships, temporal dynamics, primary cardiomyocyte models, and practical aquaculture applications. Also, the single dietary concentration tested under laboratory conditions does not establish an optimal dose or a validated commercial aquaculture feeding regimen.

## 5. Conclusions

In summary, this study infers that waterborne Mn exposure may induce oxidative stress and ER stress in carp cardiac tissue, which are associated with ISR-related changes and inflammatory responses, together with myocardial structural damage and biochemical evidence of cardiac injury. In vitro experiments using the ISR inhibitor ISRIB in EPC and H9c2 cells provide supporting evidence that ISR-related signaling may be involved in the Mn-induced inflammatory process, although these cell-based data do not constitute definitive proof of causality in carp cardiac tissue in the Mn-induced inflammatory process. Dietary EGCG supplementation attenuated these Mn-induced responses and was associated with reduced cardiac injury. These findings provide a proposed mechanistic framework for understanding heavy metal-caused cardiac injury and support further evaluation of EGCG as an experimental anti-stress dietary intervention. However, the causal chain from Mn exposure to cardiac injury through the ISR–inflammation axis remains to be fully established in vivo, and these data do not identify the responsible upstream ISR kinase or demonstrate PERK- or GCN2-specific activation. Thus, further studies with dose–response designs and in vivo cardiac functional assessments are needed to confirm these findings.

## Figures and Tables

**Table 1:** Growth performance, feed utilization, and survival of common carp in each group after the 60-day experiment.

Parameters	Con	EG	Mn	Mn + EG	*p* Value
Initial body weight (g)	76.92 ± 4.05	76.71 ± 4.18	77.03 ± 3.96	76.85 ± 4.22	>0.05
Final body weight (g)	128.54 ± 6.37	146.17 ± 5.92	127.89 ± 6.15	128.33 ± 5.86	>0.05
Total weight gain WG (g)	51.66 ± 2.41	52.29 ± 2.18	51.01 ± 2.33	51.45 ± 2.09	>0.05
Feed conversion ratio FCR	1.96 ± 0.11	1.94 ± 0.15	1.97 ± 0.13	1.95 ± 0.09	>0.05
Survival rate SR (%)	100	100	100	100	

## Data Availability

The data supporting the findings of this study are included in the article and its Supplementary Materials. Specifically, the primer sequences used for real-time PCR analysis of common carp and rat are provided in Supplementary Tables S1 and S2, respectively. The database-derived target lists are provided in Supplementary Tables S3, S8, and S9: Table S3 lists the potential EGCG-associated targets retrieved from the BATMAN-TCM 2.0 database; Table S8 lists the potential Mn-associated toxicity targets retrieved from the Comparative Toxicogenomics Database (CTD); and Table S9 lists the overlapping candidate targets between EGCG and Mn. Other supplementary tables, including enrichment analysis results (Tables S4–S7 and S10–S12), are also provided in the Supplementary Materials. Additional underlying numerical data supporting the qRT-PCR, biochemical, and other quantitative analyses are available from the corresponding author upon reasonable request.

## Supplementary Materials

The following supporting information can be downloaded at https://www.mdpi.com/article/10.3390/biology15201866/s1. Figure S1: Semi-quantitative histopathological scoring of carp heart tissues in the control, Mn, EGCG, and Mn+EGCG groups; Figure S2: The protein levels of p-eIF2α, eIF2α, ATF4, CHOP, PERK, GCN2, NF-κB, STAT3, MAPK1, MAPK14B, and JUN in H9c2 cells; Table S1: Primer sequences used for real-time PCR analysis of Common carp; Table S2: Primer sequences used for real-time PCR analysis of Rat; Table S3: The potential targets of EGCG; Table S4: KEGG pathways enrichment analysis of the EGCG potential targets; Table S5: GO enrichment analysis of the EGCG potential targets at BP level; Table S6: GO enrichment analysis of the EGCG potential targets at MF level; Table S7: GO enrichment analysis of the EGCG potential targets at CC level; Table S8: The potential toxicity targets of Mn.; Table S9: The potential EGCG targets for alleviating Mn toxicity; Table S10: KEGG pathways enrichment analysis of the potential EGCG targets for alleviating Mn toxicity; Table S11: GO enrichment analysis of the potential EGCG targets for alleviating Mn toxicity at BP level; Table S12: GO enrichment analysis of the potential EGCG targets for alleviating Mn toxicity at MF level.
