# Supplementary material for: Epigallocatechin-3-Gallate Alleviates Manganese-Induced Cardiac Injury in Common Carp via Suppression of the Integrated Stress Response–Inflammation Axis
Source: Biology (Basel). 2026 Oct 16;15(20):1866. doi: 10.3390/biology15201866 (PMC13701365; doi:10.3390/biology15201866)
Supplement: Supplementary file 1 [file biology-15-01866-s001.zip › biology-4535951-supplementary/Final-SM/Figure S1 and Figure S2.pdf]

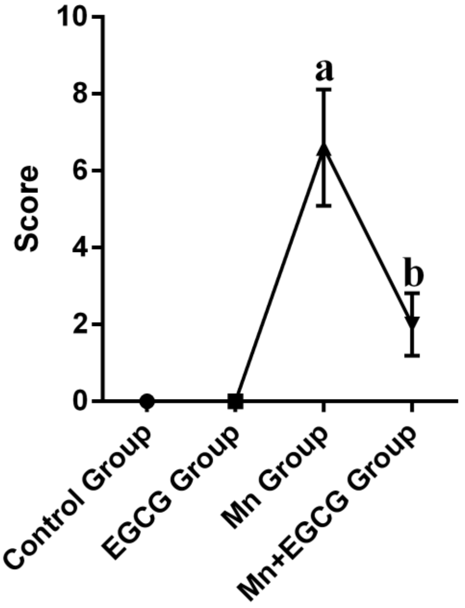

Figure S1. Semi-quantitative histopathological scoring of carp heart tissues in the control, Mn, EGCG, and Mn+EGCG groups. Three sections per group were examined. For each section, ten randomly selected microscopic fields were evaluated by an investigator blinded to group allocation. Eight parameters were scored: cardiomyocyte degeneration, cardiomyocyte necrosis, inflammatory cell infiltration, myocardial fibrosis, myocardial disarray, vascular congestion/hemorrhage, interstitial edema, and myocardial hypertrophy. Each parameter was graded from 0 to 3 (0 = normal/absent; 1 = mild; 2 = moderate; 3 = severe), and the total score was calculated as the sum of all parameters. Data are expressed as mean ± SD (n = 3). Different lowercase letters indicate significant differences between groups ($P < 0.05$).

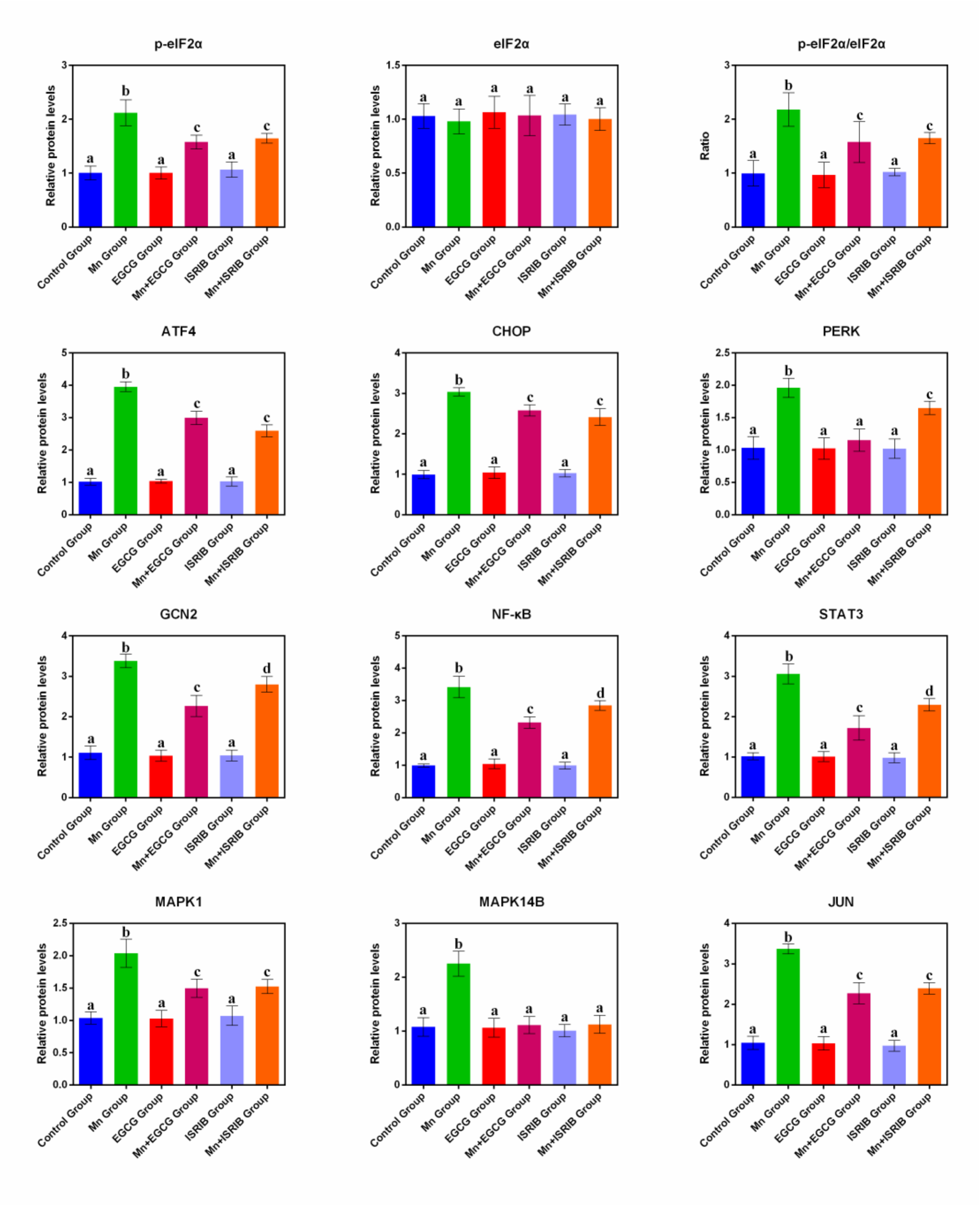

Figure S2. The protein levels of p-eIF2α, eIF2α, ATF4, CHOP, PERK, GCN2, NF-κB, STAT3, MAPK1, MAPK14B, and JUN in H9c2 cells. Data are expressed as mean ± SD (n = 3). Different lowercase letters indicate significant differences between groups ($P < 0.05$).
