# Supplementary material for: Epigallocatechin-3-Gallate Alleviates Manganese-Induced Cardiac Injury in Common Carp via Suppression of the Integrated Stress Response–Inflammation Axis
Source: Biology (Basel). 2026 Oct 16;15(20):1866. doi: 10.3390/biology15201866 (PMC13701365; doi:10.3390/biology15201866)
Supplement: Supplementary file 1 [file biology-15-01866-s001.zip › biology-4535951-supplementary/Final-SM/Table S1 and S2.pdf]

Table S1. Primer sequences used for real-time PCR analysis of Common carp

Gene name	Accession number	Primer sequence	Product size (bp)
SOD1	XM_019111527.2	Forward 5'- CTTAAAGGCACCGGGGAAGT -3'<br>Reverse 5'- TGATGCAGCCGTTTGTGTTG -3'	154
CAT	XM_042715094.1	Forward 5'- CAGCGCAGACGATGACAATG -3'<br>Reverse 5'- GTAATCGGGGTGAACAGCCA -3'	169
NRF2	XM_042763189.1	Forward 5'- TTCCCGCTGGTTTACCTTAC -3'<br>Reverse5'- CGTTTCTTCTGCTTGTCTTT -3'	158
HSPA5	XM_042752227.1	Forward 5'- ATCACCATTACCAACGACCAGAACC-3'<br>Reverse 5'- TTTCTTGTCCTCATCAGCGAATCTCTC -3'	93
DDIT3	XM_042759047.1	Forward 5'- CGCGAAAGGATGATCGGAGA -3'<br>Reverse 5'- GTGATGCGAAGCGTTGTACC -3'	152
eif2ak4	XM_042743091.1	Forward 5' - TGGCGTTTGCCAGATTTGTG -3'<br>Reverse 5'- GGCAGGAGACTGTTGCTGAT -3'	160
atf4b	XM_019077352.2	Forward 5'- CAACACCCCTTCACCCTCTC -3'<br>Reverse 5'- GGCTCAGGGACTACAGAGGA -3'	150
il1b	XM_019080073.2	Forward 5'- GCAATACAAAGTTCAATTCAGC -3'<br>Reverse 5'- GTCATTCGGGTCACCGGC -3'	196
il6	XM_019073058.2	Forward 5'- CCTGCTGGAGGCCATTAACA -3'<br>Reverse 5'- ATCGAATGCACCGTGGTCTT-3'	117
ifng1	NM_001360712.1	Forward 5'- TGGTTTGGAAAATCTGTTTTG -3'<br>Reverse 5'- TCAGCAAGCAGCGACTGAC -3'	83
nos2a	XM_042757322.1	Forward 5'- CACTGGTGCAGGCTCTTACA -3'<br>Reverse 5'- CCAAGAAGGTGGGCCTGTAG -3'	177
nfkb1	XM_042769966.1	Forward 5 - GGGCCTCCAGTGAGAAGAAC -3'<br>Reverse 5'- CCAGGCTGTGAGCATGAAGA -3'	129

stat3	XM_042720474.1	Forward 5’- GACCGAAGAACTGCACCTGA -3’<br>Reverse 5’- CCCATGCGTTTGGCATTTGA -3’	128
jun	XM_042746229.1	Forward 5’- CTCTGAAGCGCAACATGACG -3’<br>Reverse 5’- CCGTTGCTGGACTGGATGAT -3’	160
mapk1	XM_042723096.1	Forward 5’ - CATGGTTTGCTCGGCGTATG -3’<br>Reverse 5’ - GCGGATGATGTCGTTGATGC -3’	163
mapk14b	XM_042733647.1	Forward 5’ - CGCGTTGGACACAAAGTCAG -3’<br>Reverse 5’ - TTCTCTGTAGGTGCGCTTGG -3’	94
β-actin	XM_019089433.2	Forward 5’ - GATGGACTCTGGTGATGGTGTGAC-3’<br>Reverse 5’ - TTTCTCTTTCGGCTGTGGTGGTG-3’	167

Table S2. Primer sequences used for real-time PCR analysis of Rat

Gene name	Accession number	Primer sequence	Product size (bp)
SOD1	NM_017050.1	Forward 5'- AGGTTCCGAGGCCGCC -3'<br>Reverse 5'- CTTGTATTGTCCCCATATTG-3'	202
CAT	NM_012520.2	Forward 5'- ATGGCCGACAGCCGGGAC -3'<br>Reverse 5'- TGGGCCCCGCAGTCATGAT -3'	139
NRF2	XM_063284653.2	Forward 5'- GCACATCCAGACAGACACCA -3'<br>Reverse 5'- CTCTCAACGTGGCTGGGAAT -3'	168
HSPA5	NM_013083.2	Forward 5'- TCATCTGGGTGGGGAAGACT -3'<br>Reverse 5'- GGGCTCTCTTGGCCTTTTCT -3'	143
DDIT3	NM_001109986.1	Forward 5’- ATGGCAGCTGAGTCTCTGCCTTTC -3’<br>Reverse 5’- GATATAGGTGCCCCCAATTTCATC -3’	117
eif2ak4	XM_039089790.3	Forward 5’ - TGTGCCTGAGATCCTTGCAG -3’<br>Reverse 5’- TGAGGACAGCGGCGAATATC -3’	137
atf4	NM_024403.2	Forward 5'- ATGACCGAGATGAGCTTCCTG -3'<br>Reverse 5'- CAGCCCAAACCCGACTGGTC -3'	80

il1b	NM_031512.2	Forward 5'- CTGAACTCAACTGTGAAATAG -3'<br>Reverse 5'- TGGGCCGTCAGCCTCAAAGA -3'	71
il6	NM_012589.2	Forward 5'- ATGAAGTTTCTCTCCGCAAGAGAC -3'<br>Reverse 5'- GGCAGTGCTGTCAACAACATCAG -3'	71
ifng1	NM_138880.3	Forward 5’- ATGAGTGCTACACGCCGCGTC -3’<br>Reverse 5’- GACTTTCTAGGCTTTCAATGAG -3’	97
nos2a	XM_087631297.1	Forward 5’- ATGGCTTGCCCCTGGAAGTTT -3’<br>Reverse5’-TGGGAAACCATTTTGATGCTTG-3’	162
nfkb1	XM_063282630.2	Forward 5 - CAGACACCTTTGCACTTGGC-3’<br>Reverse 5’- CTTGAGTAGGACCCCGAGGA-3’	162
stat3	XM_087631394.1	Forward 5’- ATGGCTCAGTGGAACCAGC -3’<br>Reverse 5’- GCCGAGGCGCCAGGAACTG -3’	112
jun	NM_021835.3	Forward 5’- ATGACTGCAAAGATGGAAACG -3’<br>Reverse 5’- GTTGCTGTAGCCGTAGGCGCC -3’	90
mapk1	XM_006248659.6	Forward 5’- CCAAACGCTGACTCCAAAGC -3’<br>Reverse 5’- GTCGTCCAGCTCCATGTCAA -3’	174
mapk14	XM_006256142.6	Forward 5’- ATGTCGCAGGAGAGGCCC-3’<br>Reverse 5’- CGGGCACCTCCCAGACGG -3’	64
β-actin	NM_031144.3	Forward 5’- CTGGCTCCTAGCACCATGAA -3’<br>Reverse 5’- AAAACGCAGCTCAGTAACAGTC -3’	196
